# PRMT5 inhibition has a potent anti-tumor activity against adenoid cystic carcinoma of salivary glands

**DOI:** 10.1186/s13046-024-03270-x

**Published:** 2025-01-11

**Authors:** Vasudha Mishra, Alka Singh, Michael Korzinkin, Xiangying Cheng, Claudia Wing, Viktoria Sarkisova, Ashwin L. Koppayi, Alexandra Pogorelskaya, Oksana Glushchenko, Manu Sundaresan, Venkat Thodima, Jack Carter, Koichi Ito, Peggy Scherle, Anna Trzcinska, Ivan Ozerov, Everett E. Vokes, Grayson Cole, Frank W. Pun, Le Shen, Yuxuan Miao, Alexander T. Pearson, Mark W. Lingen, Bruce Ruggeri, Ari J. Rosenberg, Alex Zhavoronkov, Nishant Agrawal, Evgeny Izumchenko

**Affiliations:** 1 Department of Medicine, Section of Hematology and Oncology, University of Chicago, Chicago, IL USA; 2 Insilico Medicine, Hong Kong, China; 3 Department of Medicine, Section of Hematology and Oncology, Northwestern University, Chicago, IL USA; 4 Prelude Therapeutics, Wilmington, DE USA; 5 Department of Pathology, University of Chicago, Chicago, IL USA; 6 Department of Surgery, University of Chicago, Chicago, IL USA; 7 Ben May Department for Cancer Research, University of Chicago, Chicago, IL USA

**Keywords:** Adenoid cystic carcinoma (ACC), Protein arginine methyl transferase 5 (PRMT5), Organoid models, Patient derived xenografts (PDXs), PandaOmics, Whole exome sequencing, RNA-Seq

## Abstract

**Background:**

Adenoid cystic carcinoma (ACC) is a rare glandular malignancy, commonly originating in salivary glands of the head and neck. Given its protracted growth, ACC is usually diagnosed in advanced stage. Treatment of ACC is limited to surgery and/or adjuvant radiotherapy, which often fails to prevent disease recurrence, and no FDA-approved targeted therapies are currently available. As such, identification of new therapeutic targets specific to ACC is crucial for improved patients’ outcomes.

**Methods:**

After thoroughly evaluating the gene expression and signaling patterns characterizing ACC, we applied PandaOmics (an AI-driven software platform for novel therapeutic target discovery) on the unique transcriptomic dataset of 87 primary ACCs. Identifying protein arginine methyl transferase 5 (PRMT5) as a putative candidate with the top-scored druggability, we next determined the applicability of PRMT5 inhibitors (PRT543 and PRT811) using ACC cell lines, organoids, and patient derived xenograft (PDX) models. Molecular changes associated with response to PRMT5 inhibition and anti-proliferative effect of the combination therapy with lenvatinib was then analyzed.

**Results:**

Using a comprehensive AI-powered engine for target identification, PRMT5 was predicted among potential therapeutic target candidates for ACC. Here we show that monotherapy with selective PRMT5 inhibitors induced a potent anti-tumor activity across several cellular and animal models of ACC, which was paralleled by downregulation of genes associated with ACC tumorigenesis, including *MYB* and *MYC* (the recognized drivers of ACC progression). Furthermore, as a subset of genes targeted by lenvatinib is upregulated in ACC, we demonstrate that addition of lenvatinib enhanced the growth inhibitory effect of PRMT5 blockade in vitro, suggesting a potential clinical benefit for patients expressing lenvatinib favorable molecular profile.

**Conclusion:**

Taken together, our study underscores the role of PRMT5 in ACC oncogenesis and provides a strong rationale for the clinical development of PRMT5 inhibitors as a targeted monotherapy or combination therapy for treatment of patients with this rare disease, based on the analysis of their underlying molecular profile.

**Supplementary Information:**

The online version contains supplementary material available at 10.1186/s13046-024-03270-x.

## Introduction

Adenoid cystic carcinoma (ACC) of salivary glands is a rare, slow growing malignancy that accounts for 1–5% of all head and neck cancers [1–3] and 25–35% of salivary gland neoplasms [4, 5]. The current treatment options for primary localized ACC are restricted to surgical resection followed by radiotherapy with or without chemotherapy [6]. Unfortunately, the standard curative management has modest activity as most patients recur. This is largely due to ACC’s high tendency for perineural invasion and/or distant metastasis. Studies investigating new therapies for ACC remain challenging [7, 8], primarily due to paucity of ACC specimens and limited availability of experimental models. As a result, no FDA approved targeted approaches are currently available, making progressive ACC a difficult disease to treat [9, 10].

Although ACC display an overall low mutation burden [11], a large subset of cases (30–80% according to multiple studies) harbor chromosomal rearrangement that produce *MYB-NFIB* fusion gene, which plays a central role in the ACC pathogenesis via activation of *MYB* target genes (such as *BCL2*, *MYC*, *CD34*, and *KIT*) that are involved in regulation of cell proliferation, differentiation, apoptosis, and angiogenesis [12–15]. Activating *NOTCH1* mutations are reported in ∼ 20% of cases with recurrent and/or metastatic disease [16], and are associated with a more aggressive disease phenotype and poor overall survival (OS) as compared to patients with wild-type *NOTCH1* [17]. While these aberrations present appealing therapeutic strategies for ACC, direct targeting of MYB (a transcription factor), remains a pharmacological challenge [13, 14]. Inhibitors of NOTCH signaling [18] are still under evaluation in early stage clinical trials [19, 20] and require careful selection for patients with *NOTCH1*-activating mutations. Molecular changes in genes that encode for epigenetic modifiers (*SMARCA2*, *CREBBP*, *KDM6A*), proliferation regulators (*EGFR*, *PIK3CA*, *KRAS*, *AXL*, *MET*, *MYC*) and tumor suppressors (*ATM*, *CDKN2A*, *TP53*) have also been noted in ACC patients [11, 18, 21]. However, due to the low frequency, they do not represent attractive therapeutic opportunities, emphasizing the clinical need for the new target candidates for treatment of ACC.

Therapeutic target identification is a crucial step of the drug discovery pipeline. Artificial intelligence (AI)-driven pathway analysis algorithms applied to genomic data have recently demonstrated their efficacy in discovering novel target candidates in cancer and age-associated diseases [22–26]. In this study we applied PandaOmics TargetID, a predictive discovery engine which ranks the targets based on the scores obtained from multiple AI-powered models [25–27], on the transcriptomic data that contains 87 primary ACCs (62 sequenced internally and 25 obtained from Gene Expression Omnibus) and 35 matched tissue specific controls. Focusing on the candidates predicted to be accessible by the small molecule inhibitors, were assigned high safety ranks, and have an established protein structure, protein arginine methyl transferase 5 (PRMT5) was identified among the putative target genes with the top-scored druggability.

PRMT5 is a predominant type II protein arginine N-methyltransferase that catalyze the mono- and symmetric di-methylation of arginine residues in both histone and non-histone substrates [28]. Several studies suggest that PRMT5-induced epigenetic regulation may activate transcription of cell cycle mediators (e.g. cyclin D1 and MYC), whereas post-translationally PRMT5 methylates many signaling molecules that play a key role in critical biological processes that contribute to neoplastic transformation and chemotherapy resistance [29–34]. Overexpression of PRMT5 has been confirmed in several solid malignancies, including head and neck cancer, and is associated with poor prognosis [35–39]. Based on the wide array of cellular and transcriptional pro-oncogenic processes regulated by PRMT5, a number of inhibitors have been developed and tested in preclinical studies across several tumor types, showing promising tumor-suppressive effects as a single agent or in combination with targeted therapeutics or chemotherapy [40]. While a small pool of PRMT5 inhibitors have reached Phase I clinical trials and demonstrated some promise in patients with advanced tumors, the overall response rates to PRMT5-targeted therapy have been suboptimal [18, 26, 41, 42]. Factors hindering success include dose-limiting toxicity and incomplete understanding of the biomarkers that would guide the selection criteria for clinical trial entry.

PRT543 is an orally available PRMT5 inhibitor, which competitively binds PRMT5 at the S-adenosyl methionine (a natural cofactor of PRMT5) recognition site and inhibits its methyltransferase activity [43]. While an early signal of treatment response to PRT543 without evidence of toxicities has been observed in a subset of patients with ACC [40, 44, 45], preclinical studies investigating the effect of PRMT5 blockade remain inadequate, in part due to the limited availability of the experimental models for this rare malignancy.

Here we demonstrate that PRT543 and its brain penetrant isoform PRT811 are potent and selective PRMT5 inhibitors. Monotherapy with these agents reduces the proliferation of ACC cell lines and organoids at nanomolar concentrations, and is associated with dose-dependent decrease in *MYB* and *MYC* expression levels. Moreover, PRT543 induces significant antitumor effects in ACC patient-derived xenograft (PDX) models expressing *PRMT5* and *MYC*. Furthermore, we show that lenvatinib (the FDA approved multi-kinase inhibitor used to treat patients with advanced stage recurrent and metastatic ACC [46, 47]) enhances growth inhibitory effect of PRT811 in vitro, suggesting a potential clinical benefit for patients expressing lenvatinib favorable molecular profile. To our knowledge, this is the first study investigating the antitumor activity of PRMT5 blockade across several preclinical models of ACC. Taken together, our results pose PRMT5 as a putative target in ACC oncogenesis and provide a strong foundation for the clinical development of selective PRMT5 inhibitors as a targeted monotherapy or combination therapy for patients with ACC.

## Materials and methods

### Samples collection

Sixty-two primary ACC formalin-fixed paraffin-embedded (FFPE) samples and 16 matching normal salivary tissue specimens (adjacent non-involved tissues free of atypical cells) were collected from the Human Tissue Resource Center at the University of Chicago following Institutional Review Boards (IRB)-approved protocols. Informed written consent was obtained from all patients before sampling. All samples were reviewed by a senior pathologist to reconfirm the diagnosis and were categorized based on the predominant growth pattern seen (cribriform, tubular, or solid). Neoplasms were categorized as a solid variant when the tumor contained 30% or more solid features. Cases were also categorized by the presence or absence of high grade transformation, with features of high grade transformation including necrosis, increased mitoses, nuclear enlargement and atypia, large nests of solid growth, papillary architecture, and loss or disruption of the myoepithelial component [48]. All samples were processed for analysis by the University of Chicago Pathology Core. Lesions with a low neoplastic cellularity (< 70%) were additionally macrodissected to remove contaminating normal cells before DNA/RNA extraction.

### Cell lines and reagents

The human ACC cell line HACC2A was received from Dr Jacques Nȍr (University of Michigan) and the UFH2 cell line was received from Dr Frederic Kaye (University of Florida). Cells were monitored for mycoplasma using the MycoAlert kit (Lonza Biosciences). HACC2A cells were cultured in DMEM medium (Gibco) supplemented with 10% FBS, 200 mM L-glutamine, antibiotic-antimycotic (100X) (Gibco), 400 ng/ml hydrocortisone, 20 ng/ml epidermal growth factor, 5 µg/ml insulin and bovine brain extract (Lonza Biosciences) [18]. UFH2 cells were cultured in DMEM + GlutaMAX medium (Gibco) supplemented with 10% FBS and 5000 U/ml penicillin-streptomycin (Gibco) [18]. Authentication of cell lines was performed through STR profiling by LabCorp (Burlington, NC). PRT543 (PRT1000543-20) and PRT811 (PRT1000811-002) were obtained from Prelude Therapeutics. Lenvatinib mesylate (HY-10981 A) was purchased from MedChemExpress. All other chemicals used in this study were purchased from Sigma and prepared according to the manufacturer’s recommendations.

### Scintillation proximity based radiometric assay

Compounds were solubilized and 3-fold diluted in 100% DMSO (Sigma), following by a further dilution in the assay buffer (20mM Tris-HCl, pH 8.0, 50mM NaCl, 0.002% Tween20, 1mM TCEP, 1% DMSO) for 10-dose IC_50_ mode at a 10-fold greater concentration than the desired assay. Standard reactions were performed in a total volume of 30 µl in assay buffer, with 300nM histone H4 based AcH4-23 (Anaspec) as substrate. PRMT5/MEP50 complex was added at a final assay concentration of 2.5 nM and the compounds were allowed to preincubate for 20 min at 37 °C. The reaction was initiated by adding S-[3 H-methyl]-adenosyl-L-methionine (PerkinElmer) to final concentration of 1µM. Following a 30 min incubation at 37 °C, the reaction was stopped by adding 25µL of 8 M Guanidine HCl. 150µL of 0.3 mg/mL streptavidin YSI SPA beads suspension (Perkinelmer) in assay buffer was added to each reaction and incubated while shaking at room temperature for 30 min. The plate was centrifuged at 100 g for 30 s before reading using a MicroBeta2 scintillation counter (Perkinelmer).

### Biochemical selectivity assay

Methyltransferase selectivity was profiled at Reaction Biology Corp (Malvern, PA) in the radioisotope-based HotSpot format. Briefly, reaction mixture was prepared by mixing methyltransferase and substrate in reaction buffers. The reaction buffer for EZH1 and EZH2 was 50 mM Tris–HCl, pH 8.0, 50 mM NaCl, 1 mM EDTA, 1 mM DTT, 1 mM PMSF, and 1% DMSO. The reaction buffer for all other human methyltransferases was 50 mM Tris–HCl, pH 8.5, 50 mM NaCl, 5 mM MgCl2, 1 mM DTT, 1 mM PMSF, and 1% DMSO. The compounds were diluted in DMSO and added to reaction mixture using Labcyte Echo 550 liquid handler. Compounds were preincubated with enzymes and substrates for 20 min before 3 H-SAM was added to initiate the enzymatic reaction. The plate was incubated at 30⁰C for 1 h. The reaction was detected by a filter-binding method.

### Jump dilution assay

The PRMT5/MEP50 complex was pre-incubated with and without compound for 30 min at 37 °C to allow the formation of the enzyme-inhibitor complex (EI complex). The EI complex was then dialyzed to remove extra inhibitor. Following 1 h dialysis at room temperature, the EI complex was diluted in reaction buffer containing 1µM 3 H-labeled SAM, and 300nM of histone H4 biotinylated peptide substrate and the reaction was run for 1 h at 37 °C. During the reaction process, a small aliquot of the reaction mixture was taken out and quenched with 8 M guanidine HCl at specific time points. Streptavidin YSI SPA beads were added and the plate incubated in a MicroBeta2 (PerkinElmer) plate chamber for 1 h before reading.

### Organoid preparation

Freshly obtained surgical samples were digested first with a mixture of collagenase and dispase, followed by TrypLE (ThermoFisher). After passing through a mesh, cells were embedded in Matrigel (Corning) and cultured in four different organoid media formulations in parallel. These formulations have the same base media containing the growth factors EGF, Noggin (Sigma), R-spondin (Sigma), and FGF10 (ThermoFisher) as well as N-acetylcysteine (ThermoFisher), nicotinamide, Y-27,632 (Rho kinase inhibitor), A83-01 (TGF-β signaling inhibitor), N2, and B27 (all from Sigma). Additional components of these media include forskolin (adenylyl cyclase activator), CHIR99021 (Wnt activator), gastrin I, prostaglandin E2, FGF2, hydrocortisone, and heregulin β1 (all from Sigma). The cells that demonstrated the most robust growth were further expanded and harvested for histology, DNA and RNA isolation, or cryopreserved at low passage numbers.

### Cell viability assay

For cell lines, relative viability was determined using an Alamar Blue assay as outlined by the manufacturer (Invitrogen). New media containing 1/10 volume of Alamar Blue reagent was added to the wells and cells were incubated at 37 °C for 1 h. Fluorescence (560 nm excitation, 590 nm emission wavelengths) was measured using a BioTek fluorometer and percent viability was determined by comparing DMSO treatment to inhibitor treatment. For organoids, ATP measurements (CellTiter-Glo Luminescent Cell Viability Assays, Promega) were used to assess proliferation and viability. Medium was discarded and CellTiter-Glo 3D reagent was added to each well. After incubating 30 min at room temperature on a rotary shaker, bioluminescence activity was assessed using a plate luminometer. Cells were plated in triplicate and experiments was repeated three times.

### IncuCyte confluence assay

Relative cell confluence was determined from the microscopy images using the Incucyte live-cell analysis system as outlined by the manufacturer (Sartorius). Percent confluence was determined by comparing DMSO treated to inhibitor treated cells. Cells were plated in triplicate and repeated three times.

### Wound healing assay

Cells were cultured in triplicate in 6-well plates (1 × 10^6^ cells per well) containing culture inserts (Ibidi, Cat# 80209). On reaching 90% confluence, the inserts were removed and cells were cultured for 24 h. Gap area in individual wells were determined using ImageJ. The gap area percentage was calculated as the gap area at 24 h relative to the gap area at 0 h.

### Cell invasion assay

Cells were cultured (1 × 10^5^ cells per well) in triplicate into the upper chamber of Matrigel-coated transwell chambers (8-µm pore size, Corning, Cat# 353097) in serum-free medium, while 10% FBS medium was added to the bottom chamber to stimulate invasion. After 24 h incubation, the cells in the upper chamber were carefully removed with cotton swab and the cells that had invaded through Matrigel were stained with Differential Quick Stain Kit (EMS #26096-50), photographed at 40X magnification and quantified across three random fields.

### cDNA synthesis and reverse transcription-PCR (RT-PCR)

Total RNA was isolated using RNeasy Mini kit (Qiagen) according to manufacturer’s instructions. RNA was reverse transcribed to cDNA using High-capacity cDNA Reverse Transcription kit (ThermoFisher Scientific #4368814) and then used as a template for real-time PCR. Gene amplification was carried out on a Viaa7 real-time PCR machine (ThermoFisher Scientific) using TaqMan Gene Expression Assays (ThermoFisher Scientific). Assay IDs were: MYB (Hs00920556_m1), MYC (Hs00153408_m1), PRMT5 (Hs01047356_m1), AXL (Hs01064444_m1) and GAPDH (Hs02758991_g1). All reactions were performed in triplicate and relative RNA quantity was calculated after normalizing to GAPDH expression by the 2^−ΔΔCT^ method.

### Western blotting

Protein lysates were prepared in RIPA lysis buffer (EMD Millipore) containing 1:100 proteinase inhibitors (EMD Millipore), and protein concentrations were measured using Pierce BCA assay kit (ThermoFisher Scientific). Proteins were resolved by electrophoresis under reducing conditions in 4–12% TGX gels (BIO-RAD) according to manufacturer’s instructions and transferred to PVDF membrane (BIO-RAD). Membranes were blocked using 5% blocking grade powder in TBS-T (BIO-RAD) and incubated with primary antibodies overnight at 4 °C followed by incubation with HRP-linked secondary antibodies (BIO-RAD) for 1 h at ambient temperature. Protein bands were visualized by chemiluminescence using Clarity ECL substrate (BIO-RAD) followed by detection with Gel Doc XR System (BIO-RAD). The following primary antibodies were used: anti-SDMe-arginine (Cell Signaling Technology, 13222), anti-MYB (Cell Signaling Technology, 12319), anti-MYC (Abcam, ab32072), anti-AXL (Abcam, ab215205), anti-AKT (Cell Signaling Technology, 4691 S), anti-phospho-AKT (Cell Signaling Technology, 4060 S), anti-FGFR1 (Abcam, ab76464), anti-phosph-FGFR1 (Abcam, ab173305), and anti-GAPDH (Santa Cruz Biotechnology, sc-47724).

### Xenograft models

Early passage PDX tissues were obtained through the Adenoid Cystic Carcinoma Research Foundation (for models ACCx5M1, ACCx6, ACCx9 and ACCx11) or Champion Oncology (for model ACCx2139). All animal procedures were performed at XenoSTART (South Texas Accelerated Research Therapeutics, San Antonio) following Institutional Animal Care and Use Committee protocols. Fragments of tumor (∼ 70 mm^3^) were implanted subcutaneously into the flanks of 6–12-week-old female nu/nu athymic nude mice (The Jackson Laboratories or Charles River Laboratories). Upon reaching 150–300 mm^3^ tumor volume, mice were randomized to treatment (*n* = 5–6) or vehicle control (*n* = 5–6) groups using blinded block randomization and therapeutic dosing (chow or PO) was implemented. Tumor dimensions were measured using digital calipers blinded to the treatment group and tumor volume was calculated using the formula: width^2^ x length x 0.52. Percent mean tumor growth inhibition (%TGI) induced by PRT543 was calculated relative to the untreated control group.

### RNA sequencing analysis

The RNA Seq was performed on Illumina NovaSeq-6000 instrument. The quality of raw paired-end sequencing reads was assessed using FastQC (v0.11.7). Further, the raw reads were trimmed to remove low-quality bases and adaptor sequences using cutadapt (v3.6.dev2) with default parameters [49]. For PDXs, mouse reads were filtered out using an approach described by Callari et al. [50]. Ribosomal RNA were further removed by SortmeRNA (v4.3.4) [51]. Filtered reads were then aligned against human genome reference (hg38) and read counts per gene were calculated using STAR (v2.6.1d) [52]. To access the quality of the final aligned reads, the percent of uniquely mapped reads (above 60% ); reads mapping to multiple loci (less than 15%) and unmapped reads (less than 10% ) was considered. Fusion transcripts were identified by Star-fusion and Arriba [53, 54], followed by FISH analysis for selected samples. Raw counts pre-processing included Upper-quartile normalization and log2-transformation. Differential gene expression (DEGs) analysis has been performed using the limma-voom package inside the PandaOmics platform. Obtained gene-wise *p*-values were corrected by Benjamini–Hochberg procedure. Logarithmic fold-changes (LFC) and FDR-corrected Q-values were used to build volcano plot for differentially expressed genes (Q-value < 0.05). Then, each of the DEGs was given a status of being oncogene and(or) tumor suppressor gene following the mappings mined from OncoKB database [55, 56]. To study the dysregulation of cellular processes in ACC cases, iPANDA algorithm [57] was performed using the Reactome pathways database [58]. iPANDA calculates the activation or inhibition score for each pathway by combining precalculated gene coexpression data with gene importance factors based on the degree of differential gene expression and pathway topology decomposition. Dysregulated pathways with iPANDA score > 0.01 or < − 0.01 were considered as activated and inhibited, respectively. Gene set enrichment analysis (GSEA) was performed using the GSEAPY python package [59] using two collections of gene sets obtained from Enrichr library [60] (MSigDB_Hallmark_2020 and KEGG_2021_Human).

### Whole exome sequencing analysis

The WES was performed on Illumina NovaSeq-6000 instrument. The quality of raw paired-end sequencing reads was assessed using FastQC (v0.11.7), ensuring that the quality score was above 30 Phred scores. Picard MarkIlluminaAdapters was used to find adapters and clip them in the unmapped BAM. The pre-processing steps for read alignment and variant calling were adapted from GATK best practices pipeline [61]. Reads were aligned against the human reference genome (hg38/GRCh38) using the BWA v0.7.17 [62]. Picard toolkit v3.0.0 was used to sort reads and mark duplicates and SAMtools v1.18 was used to index BAM files. Base quality score recalibration (BaseRecalibrator, GATK v4.4.0.0) was performed using GATK with dbSNP build 138. Somatic variants were called using MuTect2 in tumor-only mode. To capture recurrent technical artifacts, GATK mutect2 was run with the panel-of-normals argument set to 1000g_pon.hg38.vcf.gz and germline resource set to af-only-gnomad.hg38.vcf.gz. Further, the raw mutations calls were filtered with FilterMutectCalls. Only variants flagged as “PASS” were retained for further analysis using snpeff v 5.1.0. ANNOVAR was used to functionally annotate variant relative to RefSeq, ensGene, gnomad211_exome, gnomad211_genome, gnomad30_genome, icgc28, cosmic92_coding, cosmic92_noncoding, dbnsfp41a annotation database [63]. For somatic mutation calling variants were filtered based on the MuTect2-assigned filter flag, tumor VAF, allele count and read depth (FILTER = PASS, VAF cutoff 5%, Allele Count Filter ≥ 3 and Depth Filter ≥ 10). Only exonic regions and non-synonymous mutations were considered for downstream analysis. All candidates were manually inspected via Integrative Genomics Viewer (IGV).

### PandaOmics TargetID target prioritization

Upper-quartile normalization and log2-transformation was applied for all transcriptomic datasets, followed by the batch correction using ComBat-seq algorithm [64]. Differential expression analysis was performed in PandaOmics using the *limma* R package. Each dataset has been processed according to *limma* standard protocols. Obtained gene-wise *p*-values were corrected by the Benjamini-Hochberg procedure. The in-silico TargetID approach was performed on the normalized transcriptomic dataset to prioritize the most promising ACC therapeutic targets from the lists of genes obtained through combining multiple ranking Omics scores (calculated based on differential expression, signaling pathway perturbation, protein–protein interaction, interactome topology, gene-disease interaction graphs, as well as gene variants curated from OMIM, ClinVar, Open Targets databases and TWAS/GWAS studies). Off note, given the limited prior knowledge about ACC, text-based scores (which represent how strongly a particular target is associated with a disease based on scientific publications, grants, patents, clinical trials and key opinion leaders) were not considered for the analysis. Detailed description of the Omic scores can be found in PandaOmics’ User Manual (https://insilico.com/pandaomics/help). The ranked list of prioritized potential targets for a disease was filtered out based on their novelty, safety, accessibility by molecule or antibodies and availability of protein data bank (PDB) structure. For this analysis, “small molecule” and “safety” filters were pre-set to include only targets that may be accessible by small compounds, don’t have any red flags in terms of safety, and have an established protein structure. “Antibody” and “novelty” filters have been used as default settings. PandaOmics TargetID druggability filters utilize traffic light logic: (a) Small molecules accessibility filter (Red - if the target does not belong to druggable protein classes and has no small molecules for it. Yellow - if the target does belong to druggable protein classes and has no small molecules for it. Green - if there is information about target druggability from at least one of informational sources such as clinical trials, therapeutic target database (TTD), open targets platform, target central resource database (TCRD)); (b) Antibody filter - ability to use antibodies to hit the target (Red - if the target is not membrane secreted [Protein atlas database]. Yellow - if the target is membrane secreted [Protein atlas database]. Green - if the target is membrane secreted [Protein atlas database] and there are antibodies in TCRD database); (c) Safety filter (Red - if the target is an essential gene and did not pass any clinical trials [Clinical trials and TTD]. Yellow - default setting for safety filter [Clinical trials and TTD]. Green - if the target is a conditional essential gene or is not essential and has clinical trials going on or passed [Clinical trials and TTD]); (d) Novelty filter (Red - more than 168 publications on pharmacognitive. Yellow − 168 or less publications on pharmacognitive. Green - less than 49 publications on pharmacognitive). After filtering, the list with the most promising hits was obtained. Detailed descriptions of all filters can be found at (https://insilico.com/pandaomics/help).

### ACC related gene signature

Genes frequently upregulated in ACC were curated from the literature and used to create a 24-gene signature (Supplementary Table 1). Only genes differentially expressed in the ACC specimens compared to the normal salivary gland samples were included.

### Statistical analysis

Student t-tests were used for statistical analysis between two groups in the in vitro experiments. For in vivo studies, natural logarithm (ln) transformation was performed on tumor volume and regression models were created for tumor volume by study day per animal. Analysis of variance was run on the slopes obtained from the regression analysis, and the Tukey–Cramer method was used to compare treatment groups. Statistical analyses were performed using GraphPad Prism software.

## Results

### Characterization of the University of Chicago (UChicago) ACC cohort

**Fig. 1:**
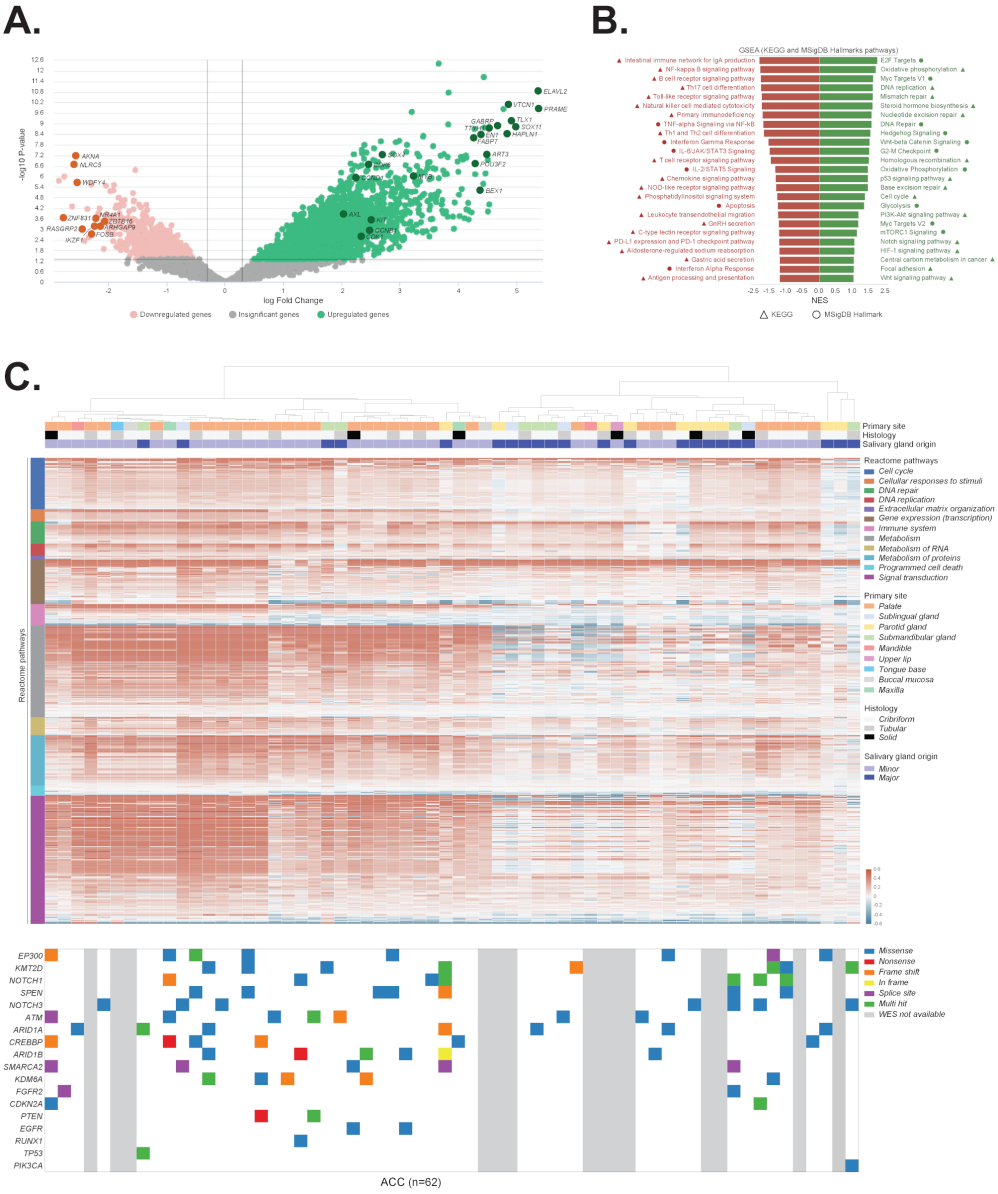
Genomic characterization of the University of Chicago ACC tumors dataset. (**A**) Volcano plot based on the transcriptomic data comparing ACC specimens and normal salivary gland samples. Green and pink colors depict significantly up-regulated and down-regulated genes respectively. Darker colors highlight genes known to be associated with ACC carcinogenesis. (**B**) Gene set enrichment analysis based on the transcriptomic data comparing ACC tumors and non-involved salivary gland samples. Analysis was performed using two collections of gene sets obtained from Enrichr library: MSigDB Hallmark 2020 and KEGG 2021 Human. Top 25 upregulated (green) and downregulated (red) pathways from MSigDB Hallmark gene sets (circle) and KEGG database (triangle) are shown. All indicated pathways have FDR q-val ≤ 0.05. (**C**) *Top*: Pathway activation heatmap comparing ACC tumors and normal salivary gland samples. Pathway activation (shades of red) or inhibition (shades of blue) is inferred based on the scores obtained from In silico Pathway Activation Network Decomposition Analysis (iPANDA) algorithm applied to Reactome pathways database. Pathways are grouped according to the Reactome’s “superpathways” that describe normal cellular functions. *Bottom*: Mutations in genes frequently mutated in ACC (reported by at least two prior studies). Multiple mutations within the same gene in a given sample are indicated once

The clinicopathologic characteristics of the 62 patients with ACC included in the UChicago cohort is summarized in Supplementary Table 2. All tumors arose in the head and neck salivary glands, with the majority (64.5%) of minor salivary gland origin and cribriform (59%) or tubular (30%) growth pattern (Supplementary Table 2). RNA-seq analysis showed that tumors in our cohort exhibited upregulation of numerous genes known to be highly overexpressed in ACC (e.g. *PRAME*, *ELAVL2*, *SOX11*, *HAPLN1*, *VTCN1*, *GABRP*, *TTYH1*, *TLX1*, *ART3*, *EN1*, *BEX1*, *POU3F2* and *FABP7*) [65–71] including drivers associated with ACC carcinogenesis such as *MYB*, *KIT*, *AXL*, *SOX4*, *CCND1*, *CCNB1* and *CDK1* (Fig. 1A, Supplementary Table 3) [69, 72–74]. The most downregulated genes contained known tumor suppressors (*ZNF831*, *AKNA*, *NR4A1*, *ARHGAP9*, *ZBTB16*) [75–79] and regulators of immune response (*NLRC5*, *WDFY4*, *RASGRP2*, *IKZF1*) (Fig. 1A, Supplementary Table 3) [80–83]. Supporting these observations, gene set enrichment analysis (GSEA) revealed that the top upregulated pathways in ACC were represented by processes that promote tumor growth, metastasis, and chemoresistance such as oxidative phosphorylation, DNA replication, cell cycle progression, glycolysis, DNA repair, MYC, WNT, NOTCH, mTORC1, Hedgehog, and PI3K-AKT signaling (Fig. 1B, Supplementary Table 4). On the other hand, pathways associated with anti-tumor immune response were enriched among the top downregulated signaling axes (Fig. 1B, Supplementary Table 4), which may explain, in part, the poor effectiveness of immunotherapies against ACC [84]. To query the transcriptomic data in more detail, iPANDA algorithm was pursued to predict differential activation of pathways retrieved from the Reactome database, which provides the hierarchical organization of signaling axes grouped into broader domains of biological functions [85]. Supporting the GSEA analysis, iPANDA revealed that signaling networks associated with cell cycle progression, gene expression, signal transduction and metabolism were upregulated in ACC, whereas cellular processes related to immune system and apoptosis were downregulated in most patients (Fig. 1C, Supplementary Table 5). Interestingly, pathway activation prediction analysis unveiled two clusters with distinct pathways activation landscape. Tumors clustering to the left side of the heatmap were enriched for palate and minor salivary gland origin (Fig. 1C), resembling the recently described ACC-I phenotype [21]. Whereas samples clustering to the right were less enriched for genetic alterations in genes frequently mutated in ACC [16, 21, 86–88] and displayed pared down activation of mitogenic signaling processes, aligning with the ACC-II malignancies (which were shown to have a less aggressive clinical course) [21, 89]. Of note, as stratification of ACC tumors into molecular subtypes is not yet sufficiently validated, and varying genetic signatures were reported to characterize patients with different clinical outcomes [21, 69, 90], these observations should be addressed with caution and require further investigation. Absence of the patient survival data and unavailability of the mutational profiles for all cases included in our cohort are the major limitations that impeded a more detailed cluster analysis.

### PRMT5 is a putative therapeutic target for ACC

**Fig. 2:**
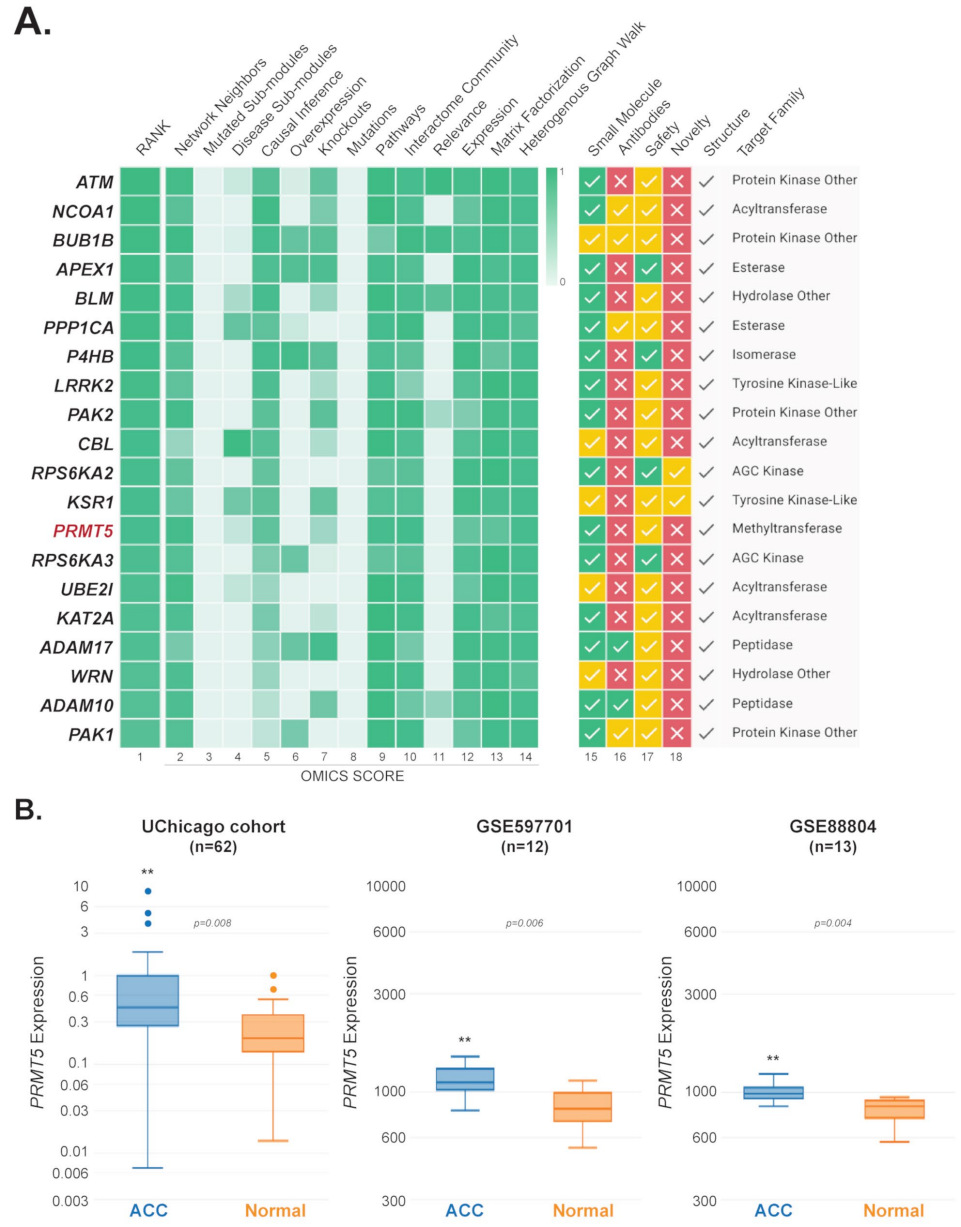
Identification of *PRMT5* as a putative therapeutic target for ACC. (**A**) The PandaOmics TargetID engine was applied to the combined ACC dataset following upper-quartile normalization and log2-transformation to rank ACC target hypotheses based on the predictive models (Omics scores) mined from the transcriptomic data. Omics score values (column 2–14) represent the probability of a given evidence group indicating the association of a given gene to a given disease. All scores range from 0 to 1, with 0 indicating no evidence, and 1 - the highest degree of evidence. The prioritized target hypotheses were screened using the Druggability filters, which utilize traffic light logic (see Methods for color keys) to indicate the most crucial protein characteristics such as accessibility by small molecules and antibodies, safety and novelty (columns 15–18). For this analysis, “small molecule” and “safety” filters were pre-set to include only targets that may be accessible by small compounds, don’t have any red flags in terms of safety, and have an established protein structure. “Antibody” and “novelty” filters have been used as default settings. A ranked list of the top 20 most promising therapeutic targets is shown. (**B**) *PRMT5* mRNA expression levels in ACC tumors and normal salivary gland tissues across three datasets used for the analysis

While effective systemic therapies for ACC are urgently needed, the process of therapeutic targets discovery represents a major challenge, especially for rare cancers such as ACC. The generative AI algorithms aimed at prediction of attention to target-disease pairs or signaling graph deconvolution are extremely helpful for efficient hypothesis generation, even with insufficient prior evidence, and offer time-efficient solutions for discovering novel target candidates for treatment of malignant diseases [26, 41, 42, 44, 91–93]. However, such methods were never applied to ACC. We thus used the PandaOmics TargetID, an AI-driven predictive target discovery engine [25–27], to identify novel putative targets against ACC. Transcriptomic profiles of patients from the UChicago cohort were combined with two datasets obtained from Gene Expression Omnibus (GSE88804 and GSE59701), the only publicly available cohorts for which non-cancerous tissue-specific control samples were available. Taken together, a single normalized meta-data used for the analysis consisted of 87 ACC samples and 35 normal controls (Supplementary Table 2). PandaOmics target discovery algorithm is based on the combination of multiple scores derived from omics data and text from scientific literature [25–27, 94], allowing to unveil the hidden hypotheses that might not be obvious from the general knowledge or simple bioinformatics analysis. However, given the limited prior knowledge about ACC in terms of publications and grants, the engine relied only on the information that was mined directly from the transcriptomic data by using Omics AI scores [25, 27, 57]. For this analysis, the main focus was given to the target expression, signaling pathways and the scores that access the interactome community of the target in protein–protein interaction (PPI) and gene-disease interaction graphs (Supplementary Fig. 1). Resulting list of targets was filtered to include proteins that belong to the druggable classes, have an established protein structure, do not have an approved inhibitors or agonists, and do not present in the Essential Gene database [95], which should ensure potential safety of a target. The list of top-scored potential targets included candidates that were reported to play tumor suppressive or oncogenic roles in several solid malignancies (Fig. 2A), including head and neck cancer (Supplementary Table 6). Although limited, prior reports indicate that a subset of the predicted targets (e.g. *ATM*, *UBE2I*, *APEX*, *ADAM1*, *BUB1B*, *PAK2*, *PRMT5*) are involved in ACC pathogenesis (Supplementary Table 6), supporting the validity of our predictive discovery pipeline. Among the genes with tumor driving properties, *PRMT5* is the only target currently under the early phase clinical trial investigations in patients with ACC [26, 41, 42]. Given the upregulated expression of *PRMT5* in ACC tumors across all independent datasets used for the analysis (Fig. 2B), its suggested role in suppression of MYC and MYB (the recognized drivers of ACC progression) [45, 96–98], and availability of selective, orally available inhibitors, we focused on evaluating the effect of PRMT5 blockade using in vitro and in vivo models of this rare malignancy.

### PRT543 is a potent and selective PRMT5 inhibitor

PRMT5 is the only PRMT that requires an obligate cofactor, methylosome protein 50 (MEP50), to function [99, 100]. To assess the potency of PRT543 to inhibit the PRMT5 enzymatic activity in a concentration-dependent manner, we have performed the in vitro scintillation proximity based radiometric assay using human recombinant PRMT5/MEP50 complex, demonstrating a strong inhibitory effect of PRT543 at nanomolar concentrations (with IC_50_ of 10.8 nM) (Supplementary Fig. 2A). Furthermore, a jump dilution experiments (assessing the time an antagonist remains bound to its target before dissociating) confirmed that PRT543 induced PRMT5/MEP50 inhibition is reversible, with a slow off-rate and long residence time (Supplementary Fig. 2B). Finally, we assessed the in vitro selectivity of PRT543 for PRMT5 compared to a panel of 36 other human methyltransferases (Supplementary Table 7). When tested at 10 µM concentration (1,000 times higher than IC_50_), PRT543 exhibited minimal (36.5%) inhibition of CARM1, with no effect on any other methyltransferase tested, indicating an exceptional selectivity (> 99% inhibition) for PRMT5 (Supplementary Fig. 2C).

### PRT543 inhibits viability of ACC cell lines and organoids

**Fig. 3:**
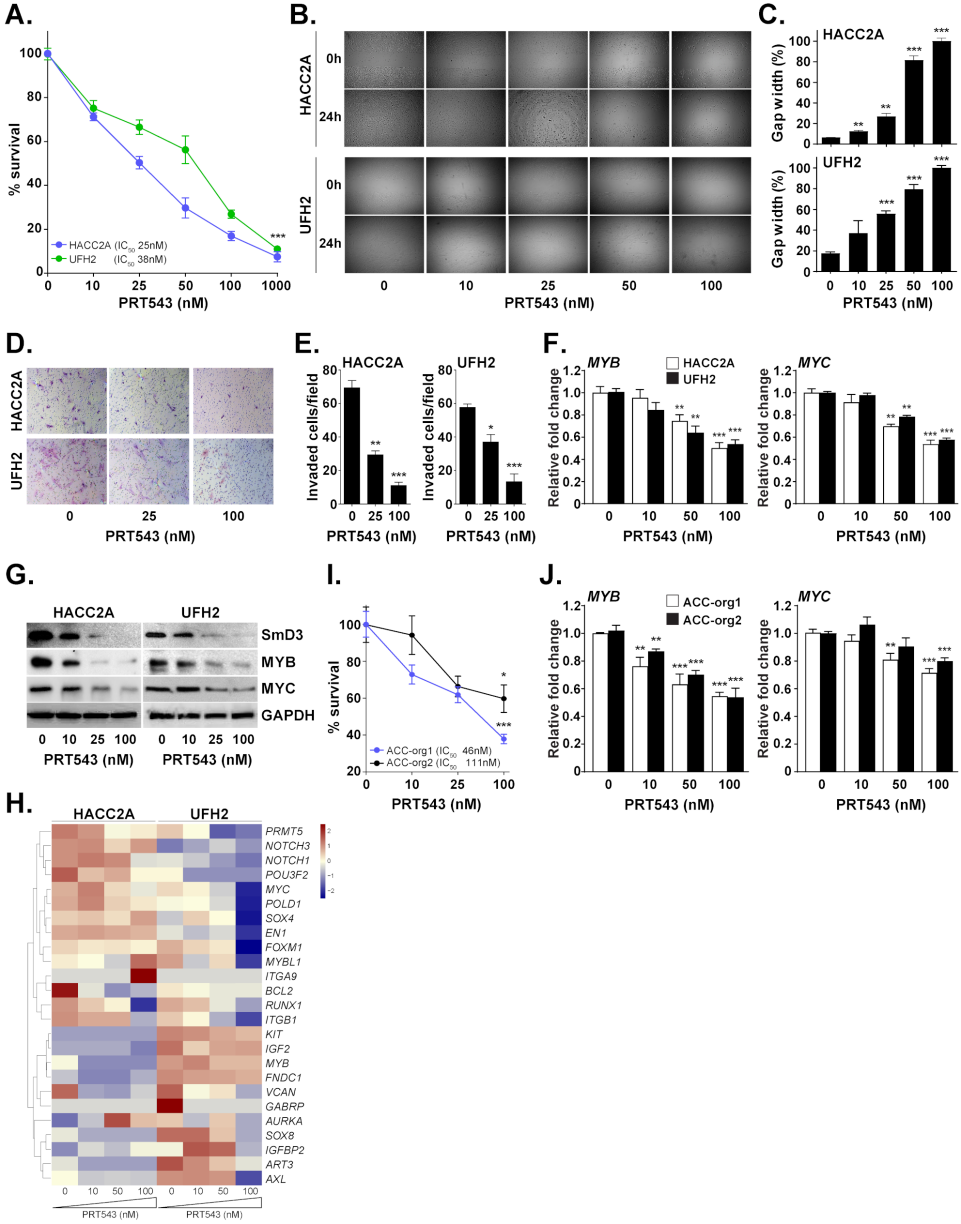
PRT543 inhibits tumorigenic properties of ACC cell lines and organoids. (**A**) ACC cell lines HACC2A and UFH2 were treated with increasing concentrations of PRT543 and relative cell viability was determined on day 7. (**B**) ACC cell lines were treated with PRT543 or DMSO for 7 days, trypsinized, counted, plated in triplicate in 6-well plates (1 × 10^6^ cells per well) containing inserts and allowed to attach for 12 h. The inserts were removed and the gap was photographed immediately and after 12 h. (**C**) Gap area in individual wells was measured using ImageJ and relative gap width percentage was calculated as the gap area at 12 h relative to the gap area at 0 h. (**D**) ACC cell lines HACC2A and UFH2 were treated with indicated concentrations of PRT543 for 7 days, trypsinized, counted and cultured (1 × 10^4^ cells per well) in triplicate into the transwell chambers. After 24 h the membranes were stained with crystal violet and cells that had migrated through the membrane were photographed. (**E**) The average number of cells per field that migrated through the membrane is shown as a bar chart. (**F**) RT-PCR analysis of *MYB* and *MYC* gene expression in cells treated with PRT543 for 7 days relative to untreated controls. (**G**) Lysates were collected at end point from cells treated with increasing concentrations of PRT543 and analyzed by western blot for the expression of indicated proteins. GAPDH was used as loading control. (**H**) Normalized expression of 24 ACC-related genes in HACC2A and UFH2 cells treated with increasing concentrations of PRT543 displayed as a heatmap. Higher or lower expression of genes is indicated with shades of red or blue cells, respectively. (**I**) Two ACC organoid models were treated with increasing concentrations of PRT543 and relative cell viability was determined on day 7. (**J**) RT-PCR analysis of *MYB* and *MYC* gene expression in organoid models treated with PRT543 relative to untreated controls. *p < 0.05, **p < 0.01, and ***p < 0.001

Due to the rarity and a slow growing nature of ACC, cellular models for this disease have rarely been established. HACC2A and UFH2 are the only naturally-immortalized, authenticated and MYB fusion expressing salivary ACC cell lines available for the analysis [18]. Treatment of these cell lines with PRT543 resulted in significantly reduced survival in a dose dependent manner, with the IC_50_ for both cells ranging in the nanomolar concentrations (Fig. 3A, Supplementary Fig. 3). Moreover, wound healing and Boyden chamber assays indicate that PRT543 significantly inhibits the migration (Fig. 3B and C) and invasion (Fig. 3D and E) abilities of both ACC cell lines compared to the untreated cells grown in parallel, supporting the role of PRMT5 in regulating the ACC tumorigenic potential in vitro. Furthermore, the effects of PRT543 treatment on cell physiology paralleled by the concentration-dependent reduction of symmetrically di-methylated SmD3, a known substrate of PRMT5 enzymatic activity, confirming that PRMT5 was functionally inhibited (Fig. 3G). Notably, treatment with PRT543 resulted in downregulation of MYB, MYC (Fig. 3F and G) and a subset of other ACC-associated genes such as transcription factors (*EN1*, *FOXM1*, *RUNX1*, *SOX8*), extracellular matrix components (*ITGB1*, *VCAN*), mitogenic regulators of cell-cycle progression (*POLD1*, *ITGB1*, *IGF2*), pro-survival oncogenes (*KIT* and *AXL*), and apoptosis suppressor (*BCL2*) (Fig. 3H), supporting the multifaceted role of PRMT5 in regulating ACC cancerogenesis [40]. Off note, while both cell lines express MYB [101, 102], the level of MYB-NFIB fusion in these cells is very low (can be only verified using nested-PCR techniques) [103] and its biologic significance remains uncertain. Compared to cell lines, organoids remain more genetically and phenotypically stable and better maintain predictive value for drug responses of individual patients. However, the use of ACC organoids remains extremely limited, with only a few models described in literature. Therefore, to study the effect of PRT543 ex vivo, we established two human organoid models internally from a surgically excised ACC tumors: ACC-org1 (bearing a confirmed *NOTCH1* activating mutation) [18], and ACC-org2 (expressing a wild type *NOTCH1*). Treatment with PRT543 significantly reduced the survival of both organoid models in a dose-dependent manner (Fig. 3I), and had potent inhibitory effects on *MYC* and *MYB* expression (Fig. 3J), the key drivers associated with ACC pathogenesis [96–98].

### PRT543 inhibits tumor growth in ACC PDX models in vivo

We next evaluated the effect of PRT543 in vivo using 5 ACC PDX models (Supplementary Table 8). The compound was administered as chow (50 mg/kg; 5 days on/2 days off) to three models (ACCx11, ACCx6, ACCx5M1) or via oral gavage (35 mg/kg; twice daily) to ACCx9 and ACCx2139 bearing mice. The dosage was selected based on the responsiveness of these ACC PDX models to various therapeutic agents (Supplementary Table 9) and PRT543 tolerability in mice bearing other tumor types [43], with higher dosage used for the PRT543 admixed in chow to ensure adequate drug intake via voluntary consumption. Notably, there were no obvious differences in drug tolerability, with both administration routes displaying similar pattern of gradual body weight loss and self-recovery (Supplementary Fig. 4). Furthermore, mice treated with PRT543 per os (ACCx9) or as chow (ACCx11) displayed significantly decreased SmD3 level, a direct readout of inhibited PRMT5 activity (Supplementary Fig. 5A), as well as reduced *MYB* and *MYC* expression (Supplementary Fig. 5B). While

**Fig. 4:**
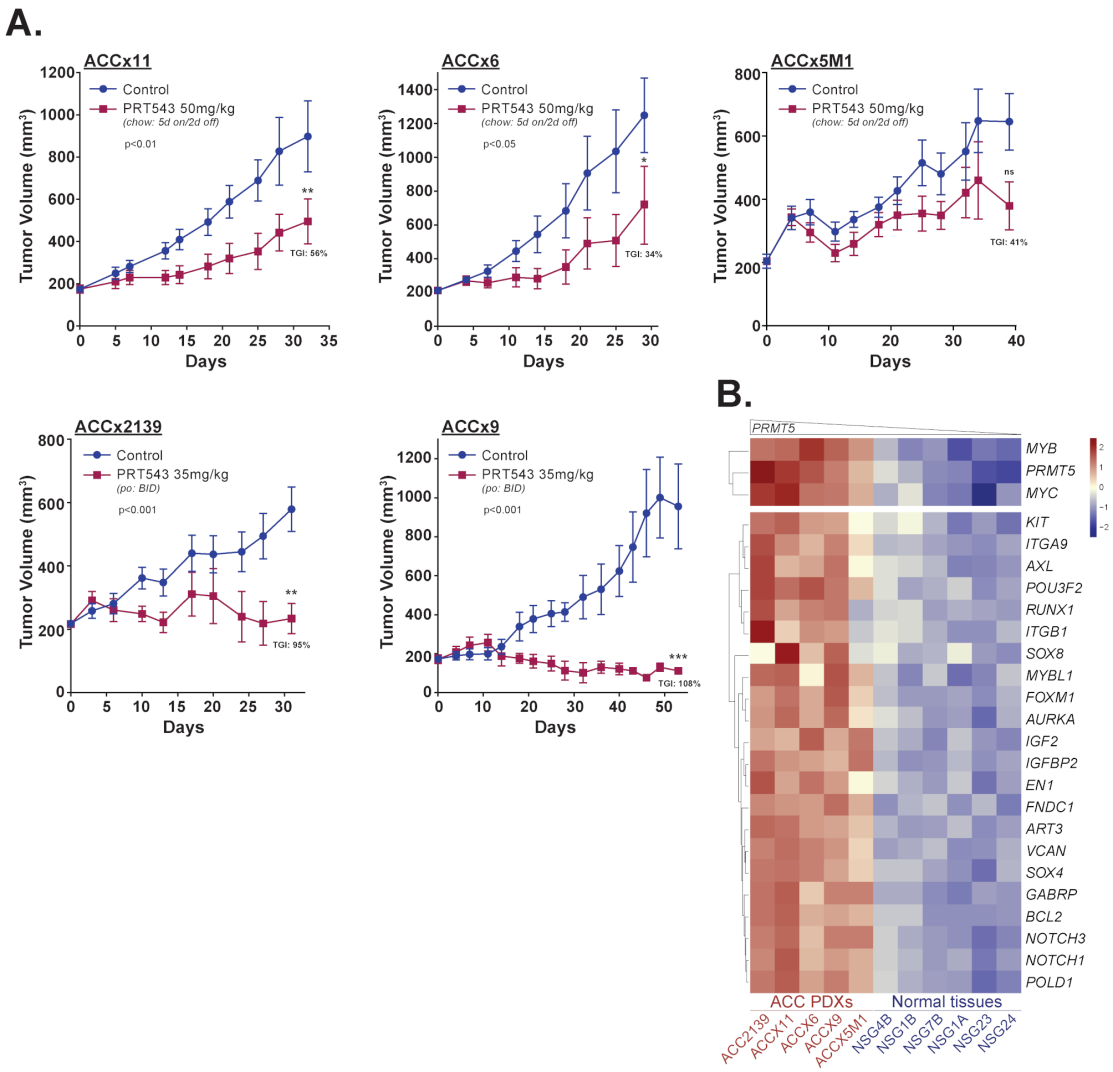
PRT543 inhibits tumor growth in ACC PDX models in vivo. (**A**) Five ACC PDX models were treated with either PRT543 (as admixed with chow for models ACCx11, ACCx6 and ACCx5M1 or po for models ACCx2139 and ACCx9) or vehicle. Graphs show the average tumor volume for 5–6 animals ± SD. Red arrow: dosage reduced on day 28 and administered at 5 mg/kg. *p < 0.05, **p < 0.01, ****p < 0.001, ns - not significant. (**B**) Hierarchical clustering of normalized baseline expression of 24 ACC-related genes in 5 ACC PDX models and 6 normal human salivary gland tissues displayed as a heatmap. Higher or lower expression of genes is indicated with shades of red or blue cells, respectively

PRT543 therapy induced significant tumor growth inhibition (TGI) in ACCx9, ACCx11, ACC2139 and ACCx6 PDXs, limited effect was seen in ACCx5M1 model (Fig. 4A). Interestingly, RNA sequencing analysis of the PDXs and normal human salivary gland tissues revealed that ACCx5M1 model displays the lowest baseline expression of *PRMT5*, *MYC* and a subset of other ACC-related genes (Fig. 4B), suggesting their potential role of in mediating the PRT543 induced antitumor activity.

### PRT811, a brain penetrant PRMT5 inhibitor, mimics the anti-proliferative effects of PRT543 in vitro

**Fig. 5:**
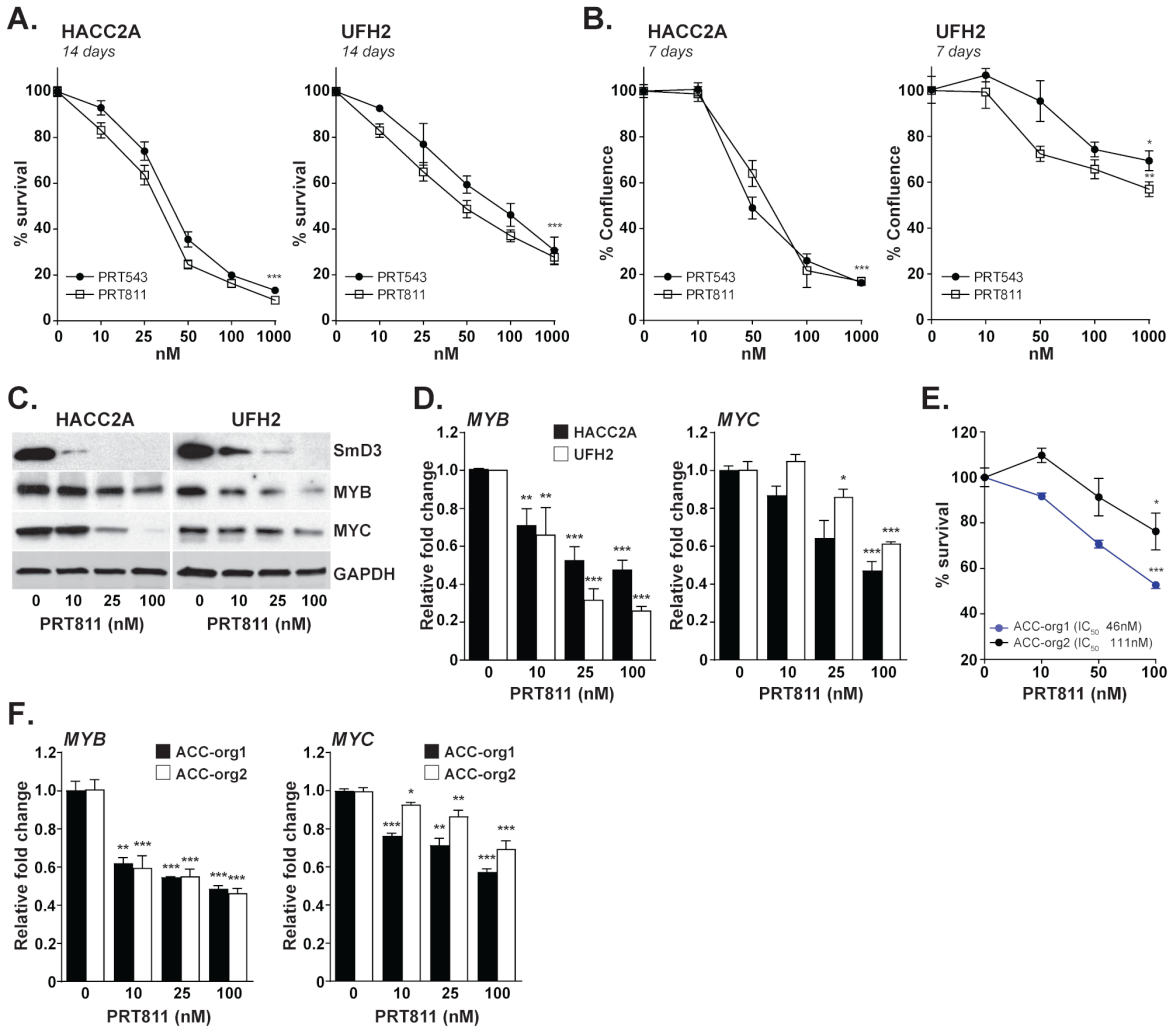
PRT811, a brain penetrant PRMT5 inhibitor, mimics the anti-proliferative effects of PRT543 in vitro. (**A-B**) Two ACC cell lines were treated with the indicated concentrations of either PRT543 or PRT811. Relative cell viability (**A**) and proliferation (**B**) were assessed at the end point. (**C**) Lysates were collected from cells treated with increasing concentrations of PRT811 for 7 days and analyzed by western blot for the expression of indicated proteins. GAPDH was used as loading control. (**D**) RT-PCR analysis of *MYB* and *MYC* gene expression in cell lines treated with PRT811 for 7 days relative to untreated controls. (**E**) Two ACC organoid models were treated with increasing concentrations of PRT811 and relative cell viability was determined on day 7. (**F**) RT-PCR analysis of *MYB* and *MYC* gene expression in organoid models treated with PRT811 relative to untreated controls. *p < 0.05, **p < 0.01 and ***p < 0.001

To further confirm that the phenotype observed in ACC cells treated with PRT543 indeed resulted from a direct inhibition of PRMT5 activity, we treated the cell lines and organoid models with PRT811, another potent, highly selective (Supplementary Fig. 6), and orally bioavailable brain penetrant PRMT5 inhibitor [104]. Similarly to PRT543, treatment with PRT811 induced a significant inhibition of cell viability (Fig. 5A and B), paralleled by a dose dependent decrease in MYB, MYC and SmD3 levels (Fig. 5C and D). Experiments carried out in two organoid models also resulted in substantially decreased viability and downregulation of *MYB* and *MYC* mRNA expression (Fig. 5E and F), further supporting the role of PRMT5 in regulating the tumorigenic potential of ACC cells in vitro.

### Lenvatinib enhances the anti-proliferative effects of PRMT5 inhibition in vitro

**Fig. 6:**
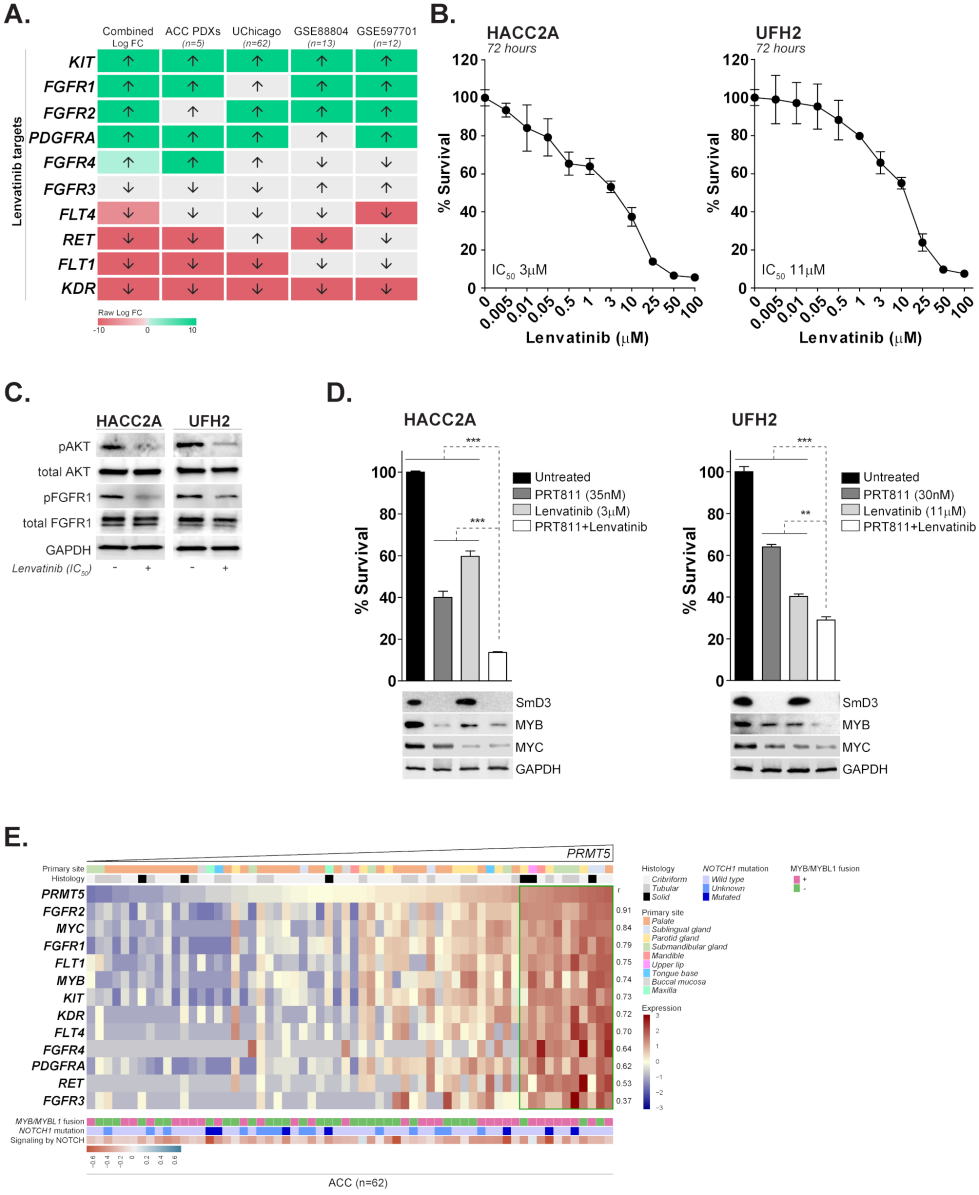
Lenvatinib enhances the anti-proliferative effects of PRMT5 inhibition in vitro. (**A**) mRNA expression level of tyrosine kinases targeted by lenvatinib was analyzed across the indicated datasets. The up pointing and down pointing arrows indicate upregulated and downregulated gene expression, respectively, relative to the normal controls. Differentially upregulated or downregulated genes are indicated with shades of green or red cells, respectively. Gray color indicate genes whose expression is not significantly altered (*p*-value > 0.05) relative to the normal controls. (**B**) ACC cell lines HACC2A and UFH2 were treated with increasing concentrations of lenvatinib for 72 h. At the end point, relative cell viability was determined and IC_50_ value for each cell line was calculated. (**C**) Lysates were collected from HACC2A and UFH2 cells treated with the IC_50_ concentration of lenvatinib for 72 h and the expression of indicated proteins was analyzed by western blot. (**D**) HACC2A and UFH2 cell lines were treated with the IC_50_ concentrations of PRT811 for 7 days, following by the exposure to IC_50_ concentrations of lenvatinib at days 8–10. Relative cell viability was determined at the end point (*top*), and lysates were analyzed by western blot for the expression of indicated proteins (*bottom*). GAPDH was used as loading control **p < 0.01 and ***p < 0.001. (**E**) The *PRMT5* mRNA expression in 62 primary ACC tumors is sorted from low to high. The corresponding expression of *MYC*, *MYB* and lenvatinib target genes are shown for each patient. Higher or lower expression of genes is indicated with shades of red or blue cells, respectively. Pearson coefficient indicates correlation between levels of each gene displayed on the heatmap with *PRMT5* mRNA expression. Green frame - a subset of tumors with highest expression of *PRMT5* is enriched for elevated levels of *MYC*, *MYB* and lenvatinib target genes

Lenvatinib is a multitargeted tyrosine kinase inhibitor with a potent suppressive activity on several pro-oncogenic targets such as VEGFR, FGF, PDGFRα, KIT and RET. Given the proposed relevance of several of these kinases to ACC biology [105], lenvatinib monotherapy was evaluated in a few early stage clinical trials, demonstrating encouraging activity against biomarker unselected recurrent/metastatic ACC, albeit with significant treatment-related toxicity [46, 47]. Analysis on the 3 ACC transcriptomic datasets and PDX models reveled that angiogenesis regulators (*FLT1*, *KDR*, and *FLT4*) are largely downregulated in ACC (Fig. 6A). Whereas expression of lenvatinib targets that play role in survival and mitogenic processes such as *KIT*, *FGFR1*, *FGFR2*, and *PDGFRA*, was elevated in ACC tumors compared to the normal salivary gland epithelia (Fig. 6A), suggesting that inhibition of their activity may potentially enhance the anti-proliferative effect of PRMT5 blockade. To explore the potential anti-proliferative effect of a combination therapy in vitro, we first identified the IC_50_ values for lenvatinib induced growth inhibition in HACC2A and UFH2 cell lines (Fig. 6B and C). Cells were then pre-treated with IC_50_ concentrations of PRT811 for 7 days, following by treatment with lenvatinib (at IC_50_ concentrations) for additional 72 h (Supplementary Fig. 7). In both cell lines, the combination resulted in a more potent growth inhibitory effect compared to the single agent therapy, and was paralleled by the strong reduction of MYB and MYC expression levels (Fig. 6D). Interestingly, a subset of patients included in the UChicago dataset displayed high mRNA expression of *PRMT5* along with elevated levels of *MYC*, *MYB* and lenvatinib target genes (Fig. 6E), suggesting that combination of inhibitors targeting PRMT5 signaling with lenvatinib may provide a potential intervention strategy for patients carrying such expression pattern. Notably, the observed pattern was not associated with NOTCH1 mutational status (Supplementary Table 10) and didn’t show a clear correlation with MYB/MYBL1 fusion gene presence or histological site. While intriguing, follow up studies using in vivo models are warranted to confirm this observation and assess the combination treatment for tolerability and efficacy.

## Discussion

Current treatment options for ACC of the salivary glands are limited to surgical excision and adjuvant radiotherapy. No standard chemotherapy or FDA-approved targeted therapy for ACC currently exists and many patients suffer from recurrent and/or metastatic disease [105]. Due to its rarity, molecular mechanisms that govern a pathogenesis of this slow growing, but relentless malignancy remain poorly understood. As such, development of safe and effective therapies for patients with ACC is imperative [84].

A comprehensive genomic profiling of ACC is crucial for identification of actionable therapeutic targets for drug discovery and precision therapy. In recent years, a tremendous growth has been observed in exploring AI-driven algorithms as a promising approach for the identification of putative therapeutic target candidates for cancer and age-associated diseases [25, 26, 57, 94, 106–108]. Our group and others have demonstrated that advanced AI algorithms applied to transcriptomic data can identify targets and biomarkers even when prior evidence is sparse [27, 57, 109, 110]. However, given the limited sequencing data available for ACC, discovery of effective therapeutic targets represents a major clinical challenge.

To navigate this limitation we harnessed PandaOmics, an AI based predictive target discovery engine [25–27, 57, 94], on a transcriptomic dataset of 87 primary ACCs, discovering *PRMT5* [28] among the top-scored putative therapeutic targets after applying novelty, small molecule druggability, and protein class filtering. Recently it was suggested that PRMT5-induced epigenetic regulation may activate the expression of *MYC* [111–113] and *MYB* [28, 96] (the key molecular drivers of ACC progression) [96–98], further supporting its role as putative therapeutic candidate for ACC. While a small number of PRMT5 inhibitors have reached phase-I clinical trials in patients with advanced malignancies [18, 26, 41, 42, 45], responses have been suboptimal [40, 45], and the preclinical studies investigating the efficacy of PRMT5 blockade in ACC are still lacking, in part due to the scarcity of experimental model systems. Here we used novel PRMT5 blockers coupled with a panel of disease specific cellular and animal models to better understand its functions and potential as a therapeutic target in ACC, emphasizing the importance of pre-selecting the patients based on their underlying molecular profile in order to improve the efficacy of the treatment.

PRT543, a potent orally available PRMT5 inhibitor [43], has demonstrated an early signal of anti-tumor activity in a subset of patients with several solid and hematological cancers, including ACC [45, 114, 115]. Our studies indicate that PRT543 selectively binds to PRMT5 and strongly inhibits its methyl-transferase activity at nanomolar concentrations, resulting in a significant dose-dependent anti-proliferative effect in two authenticated ACC cell lines and two ACC human organoid models, which was paralleled by downregulation of *MYB*, *MYC* and a panel of ACC-associated genes. Notably, PRMT5 inactivation by either PRT543 or PRT811 (another highly selective, brain penetrant PRMT5 inhibitor) resulted in a comparable inhibitory profile in organoid models with wild-type or mutated *NOTCH1*. This was supported by our in vivo analysis (demonstrating that PRT543 induced anti-tumor activity in PDXs irrespective of *NOTCH1* alteration status), and further confirmed by the recent phase-I clinical trial that assessed safety and efficacy of PRT543 in patients with recurrent/metastatic ACC [45]. While these observations suggest that in ACC PRMT5 may regulate signaling processes downstream of NOTCH1, further mechanistic studies are needed to support or refute this suggestion.

Although PRT543 and JNJ-64619178(another PRMT5 inhibitor under early clinical investigation) had a favorable toxicity profile and showed preliminary evidence of antitumor activity in patients with advanced ACC, the observed efficacy was limited [45, 116]. While this may be explained by enrolment of patients with progressive disease that were subjected to multiple lines of therapy prior to receiving the investigational anti-PRMT5 treatment, these results also emphasize that biomarkers predictive of responsiveness to PRMT5 inhibition are needed to further improve outcomes of patients treated with this therapeutic strategy. Unfortunately, such biomarkers have not yet been identified. The correlation between splicing factor mutations and efficacy of PRMT5 inhibitors observed in select myeloid malignancies [117, 118] was not confirmed in solid tumors, including ACC [45, 117]. Recent preclinical and clinical evidence suggest that methylthioadenosine phosphorylase (MTAP)-null selective PRMT5 inhibitors (e.g. AMG193, MRTX1719 and TNG908) may induce partial responses in ∼10% of patients with variety of MTAP deficient tumors [119–121]. However, such alterations are infrequent in ACC [122]. Our data suggest that high level of *PRMT5* and *MYC* expression is associated with better response to PRMT5 blockade, providing a rationale for further validating this association in prospective clinical trials.

As PRT543 was generally well tolerated by patients with recurrent/metastatic ACC [45], the anti-tumor activity of PRMT5 inhibitors may be further enhanced when given in combination with other therapeutic agents. Among systemic therapies, several multi-kinase inhibitors that target RAF/MEK/ERK pathway in tumor cells and VEGFR tyrosine kinases in tumor vasculature, have demonstrated promising clinical benefit in patients with ACC [8, 46, 123, 124]. Currently, the national comprehensive cancer network guidelines identify three multi-kinase inhibitors (axitinib, sorafenib, and lenvatinib) as “useful in certain circumstances” for ACC in the recurrent/metastatic setting [8]. While early studies have demonstrated VEGF overexpression in a subset of surgically resected tumors [125, 126], expression of VEGF receptors in ACC was rarely assessed or compared to the non-tumorous tissues [127]. Our results indicate that mRNA expression of *FLT1*, *KDR*, and *FLT4* (genes that encode for VEGFR1, VEGFR2 and VEGFR3 respectively) is lower in ACC tumors compared to the normal salivary gland epithelia, which may explain the modest effect of anti-angiogenesis therapy in unselected patients with ACC. In contrast to VEGFRs, expression of *KIT*, *FGFR1*, *FGFR2*, *FGFR4* and *PDGFRA* (additional targets of a small-molecule tyrosine kinase inhibitor lenvatinib associated with oncogenesis and maintenance of the tumor microenvironment in several types of cancer) was largely elevated among ACC tumors analyzed in this study. While our in vitro results coupled with the transcriptomic analysis of ACC tumors suggest that a subpopulation of patients that demonstrates high *PRMT5* expression along with elevated levels of *MYC*, *MYB* and direct targets of lenvatinib may benefit from the treatment that combines lenvatinib and PRT543, further validation in preclinical animal models and clinical settings is required to assess the feasibility of this putative therapeutic strategy.

## Conclusion

Taken together, our study demonstrates the antitumor activity of PRT543 in several preclinical models of ACC, providing a strong rationale for further investigation of PRMT5 inhibition as a targeted monotherapy or combination therapy for a subset of patients with ACC based on the analysis of their underlying molecular profile.

## Electronic supplementary material

Below is the link to the electronic supplementary material.

Supplementary Figure 1: Overall pipeline of the PandaOmics TargetID approach. Transcriptomic data from the UChicago cohort was combined with two publicly available ACC datasets (GSE88804 and GSE59701) into a single Meta-analysis that was passed into the PandaOmics prioritization engine for target discovery. Given the limited prior knowledge about ACC, we utilized only the set of 13 AI-based Omics scores for the target search. Ranked list of target candidates was filtered with respect to the protein class, druggability assessment, target development level and tissue specificity

Supplementary Figure 2: (**A**) Concentration-dependent inhibition of PRMT5/MEP50 enzymatic activity by PRT543 in a scintillation proximity based radiometric assay. Data represent mean ± SD. (**B**) Jump dilution assay showing PRMT5/MEP50 enzymatic progress curve in the absence and presence of PRT543. CPM - counts per minute. (**C**) Biochemical selectivity of PRT543 against 37 human methyltransferases. Percent control represents % enzymatic activity remaining in the presence of 10 µM PRT543 relative to DMSO control

Supplementary Figure 3: HACC2A and UFH2 cells were treated with increasing concentrations of PRT543 for 7 days and grayscale microscopy images were taken

Supplementary Figure 4: Graphs show the average body weights for the PDX models used in this study that were treated with either PRT543 (red curves) or vehicle (blue curves)

Supplementary Figure 5: (**A**) ACCx9 and ACCx11 tumor tissues were harvested from PRT543 or vehicle treated mice, lysates were collected and analyzed by western blot for the expression of SmD3. β-actin was used as loading control. (**B**) RT-PCR results demonstrating significant reduction of *MYB* and *MYC* gene expression in PRT543 treated ACCx9 and ACCx11 PDX models relative to the control animals (red line)

Supplementary Figure 6: (**A**) Concentration-dependent inhibition of PRTMT5/MEP50 enzymatic activity by PRT811. (**B**) Jump dilution assay showing PRMT5/MEP50 enzymatic progress curve in the absence and presence of PRT811. CPM - counts per minute. (**C**) Biochemical selectivity of 10µM PRT811 against 37 human methyltransferases. Percent control represents % enzymatic activity remaining in the presence of PRT811 relative to DMSO control

Supplementary Figure 7: Schematic presentation of the experiment. HACC2A and UFH2 cell lines were treated with the IC_50_ concentrations of PRT811 for 7 days, following by the exposure to IC_50_ concentrations of lenvatinib at days 8–10

Supplementary Table 1: List of genes included in the ACC related gene signature

Supplementary Table 2: Demographic and clinical characteristics of patients with primary ACC used in this study

Supplementary Table 3: Significantly differentially expressed genes between ACC tumors and normal samples. Genes with LFC ≥ 1.5 and ≤ -1.5 were included. Genes labeled in green (top of the list) and orange (bottom of the list) are highlighted on the Volcano plot

Supplementary Table 4: MSigDB Hallmark and KEGG pathways, sorted by the normalized enrichment score (NES), used in GSEA analysis of the transcriptomic data from ACC tumors and normal specimens. Top 25 upregulated pathways (labeled in red at the top of the list) and top 25 downregulated pathways (labeled in green at the bottom of the list) are depicted in Fig. 1B

Supplementary Table 5: Significantly dysregulated Reactome pathways resulting from the iPANDA transcriptomic analysis of the ACC tumors and normal specimens included in the UChicago cohort

Supplementary Table 6: List of the top-ranked candidate ACC target genes

Supplementary Table 7: List of 37 methyltransferases included in the in vitro selectivity assays

Supplementary Table 8: Characterization of ACC PDX models used in this study

Supplementary Table 9: Baseline in vivo responsiveness to various therapeutic agents in ACC PDX models (data obtained from the ACC Research Foundation)

Supplementary Table 10: *NOTCH1* mutations identified across ACC tumors included in the UChicago dataset

## Data Availability

The authors declare that the data supporting the findings of this study are available within the article and its supplementary information files. Source data are provided with this paper. Patients’ de-identified data (such as gender, age, tumor site and grade) is provided in the manuscript. Raw RNA-Seq data have been uploaded to the NCBI Sequence Read Archive repository (PRJNA1200774).

## Abbreviations

ACC: Adenoid cystic carcinoma
OS: Overall survival
PDX: Patient-derived xenograft
DEG: Differential gene expression
FFPE: Formalin-fixed paraffin-embedded
GSEA: Gene set enrichment analysis
PPI: Protein–protein interaction
MEP50: Methylosome protein 50
TGI: Tumor growth inhibition
MTAP: Methylthioadenosine phosphorylase
AI: Artificial intelligence
PRMT5: Protein arginine methyl transferase 5
TCRD: Target central resource database
TTD: Therapeutic target database
iPANDA: In silico pathway activation network decomposition analysis
