# Supplementary figures and images for: PRMT5 inhibition has a potent anti-tumor activity against adenoid cystic carcinoma of salivary glands
Source: J Exp Clin Cancer Res. 2025 Jan 11;44:11. doi: 10.1186/s13046-024-03270-x (PMC11724466; doi:10.1186/s13046-024-03270-x)

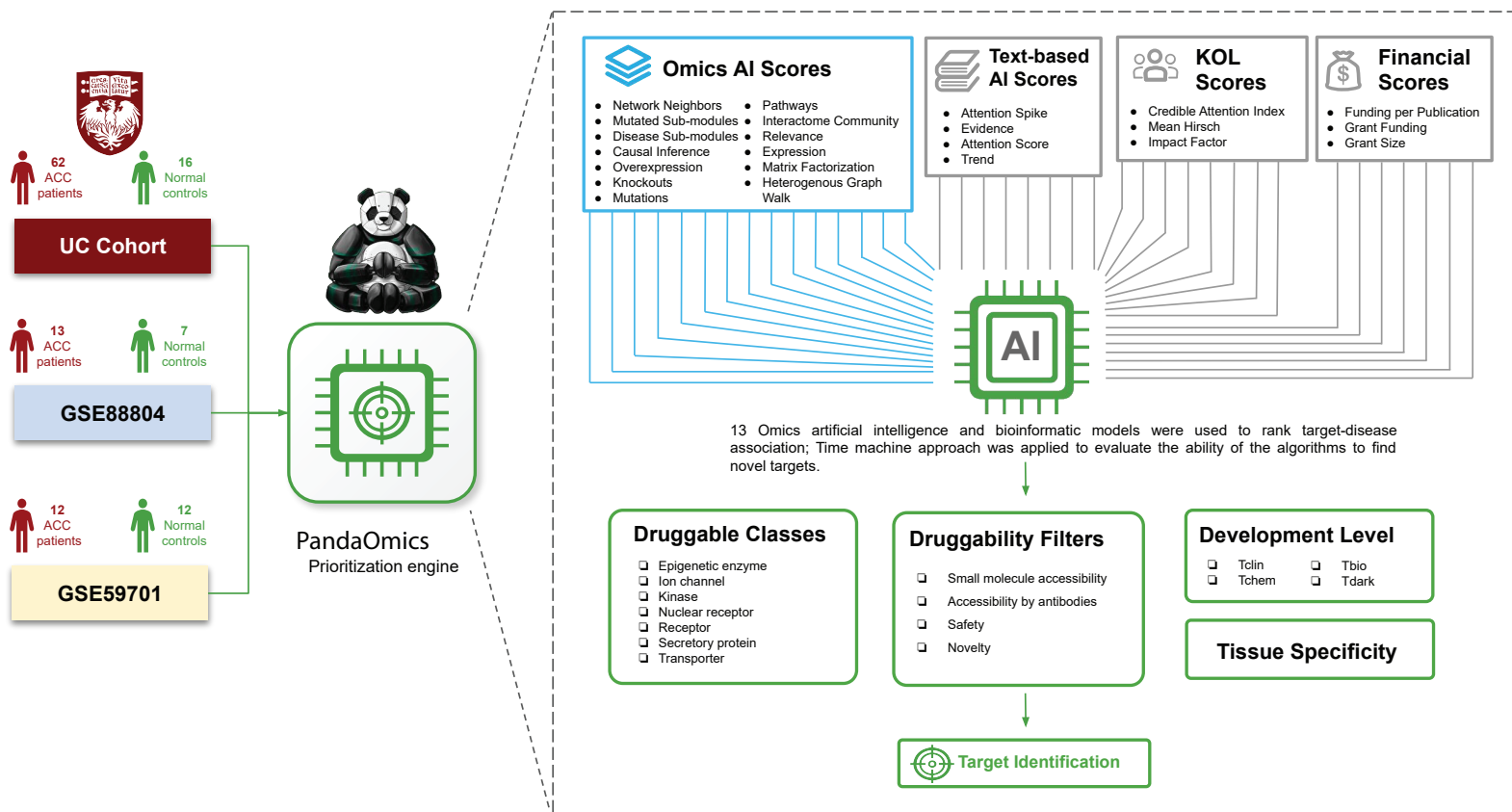

**Supplementary Figure 1**

Supplement: Supplementary file 1 — Supplementary Figure 1: Overall pipeline of the PandaOmics TargetID approach. Transcriptomic data from the UChicago cohort was combined with two publicly available ACC datasets (GSE88804 and GSE59701) into a single Meta-analysis that was passed into the PandaOmics prioritization engine for target discovery. Given the limited prior knowledge about ACC, we utilized only the set of 13 AI-based Omics scores for the target search. Ranked list of target candidates was filtered with respect to the protein class, druggability assessment, target development level and tissue specificity [file 13046_2024_3270_MOESM1_ESM.pdf]

**A.**

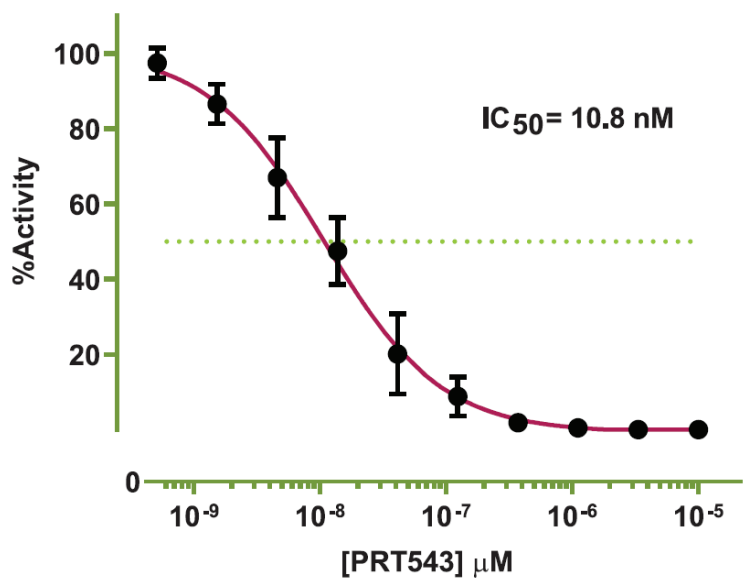

**B.**

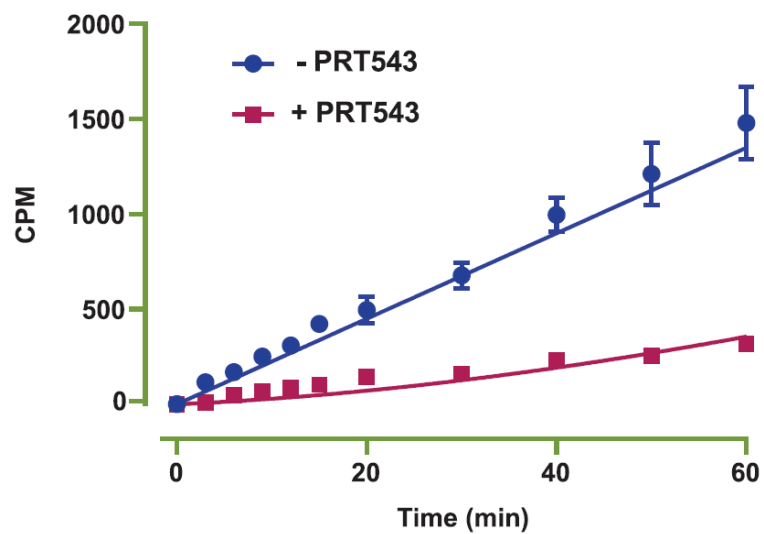

**C.**

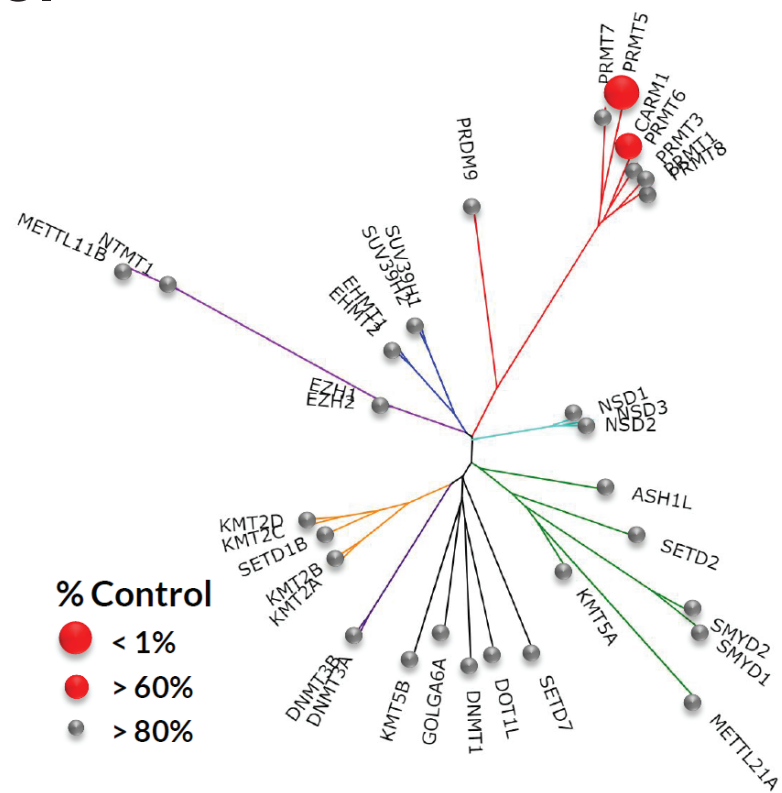

**Supplementary Figure 2**

Supplement: Supplementary file 2 — Supplementary Figure 2: (A) Concentration-dependent inhibition of PRMT5/MEP50 enzymatic activity by PRT543 in a scintillation proximity based radiometric assay. Data represent mean ± SD. (B) Jump dilution assay showing PRMT5/MEP50 enzymatic progress curve in the absence and presence of PRT543. CPM - counts per minute. (C) Biochemical selectivity of PRT543 against 37 human methyltransferases. Percent control represents % enzymatic activity remaining in the presence of 10 µM PRT543 relative to DMSO control [file 13046_2024_3270_MOESM2_ESM.pdf]

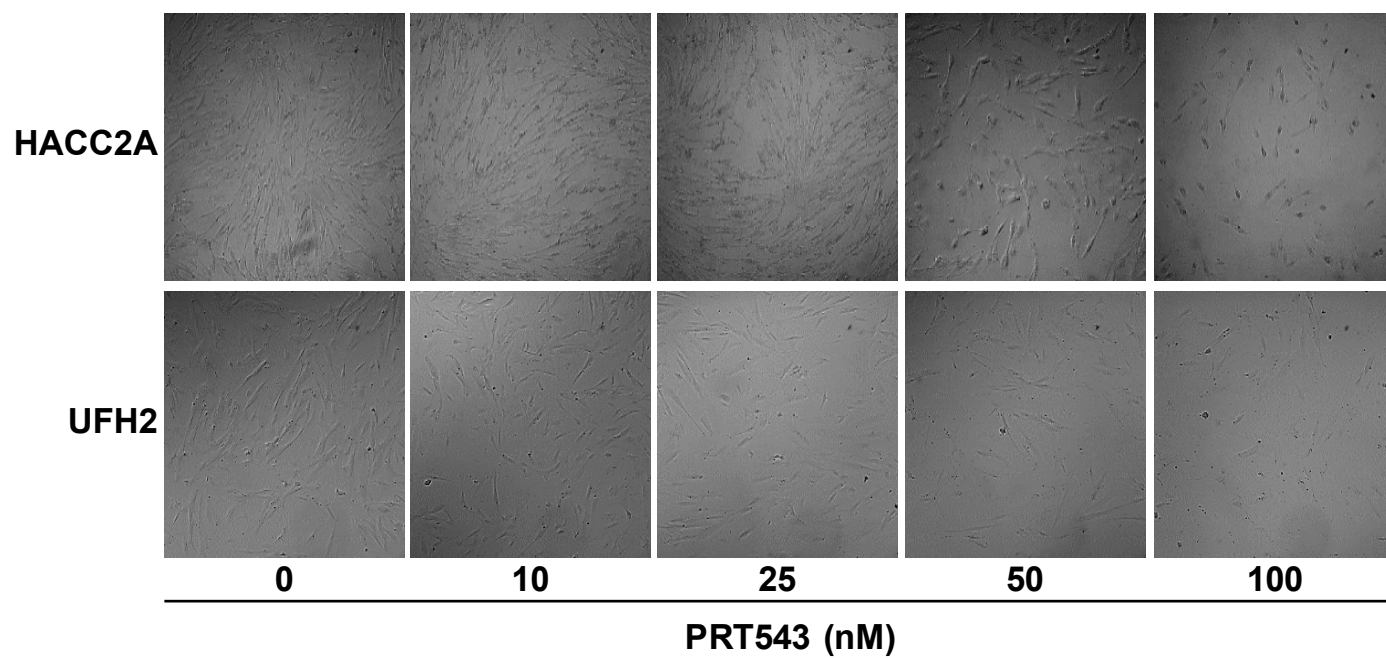

**Supplementary Figure 3**

Supplement: Supplementary file 3 — Supplementary Figure 3: HACC2A and UFH2 cells were treated with increasing concentrations of PRT543 for 7 days and grayscale microscopy images were taken [file 13046_2024_3270_MOESM3_ESM.pdf]

**A.**

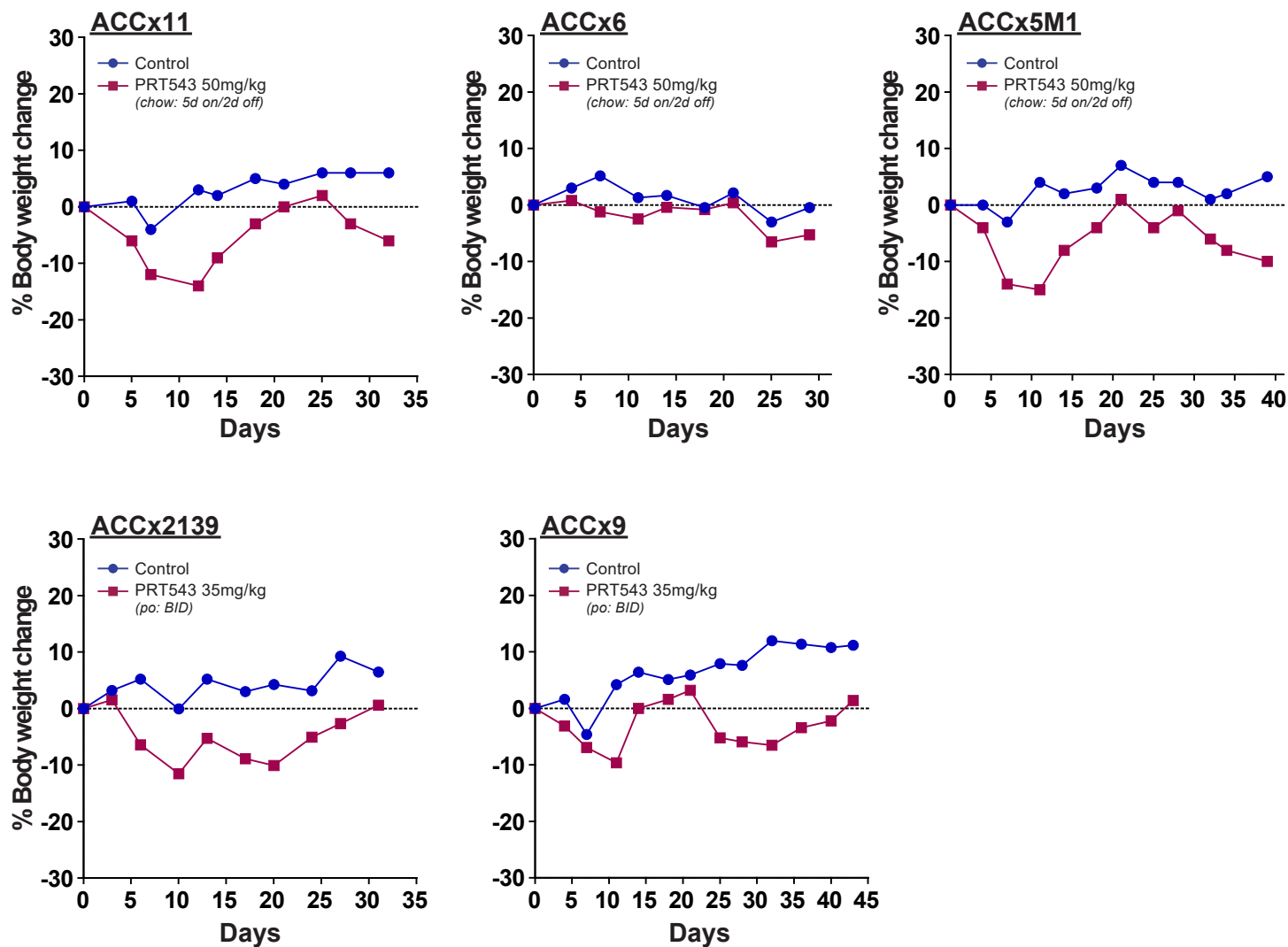

**Supplementary Figure 4**

Supplement: Supplementary file 4 — Supplementary Figure 4: Graphs show the average body weights for the PDX models used in this study that were treated with either PRT543 (red curves) or vehicle (blue curves) [file 13046_2024_3270_MOESM4_ESM.pdf]

A.

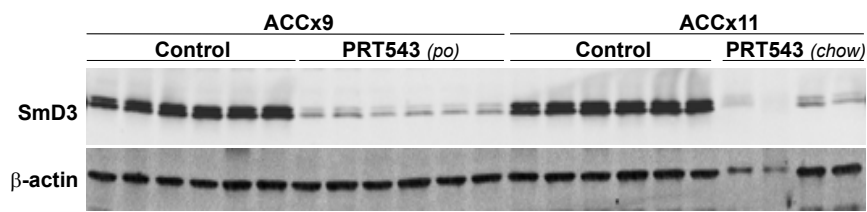

B.

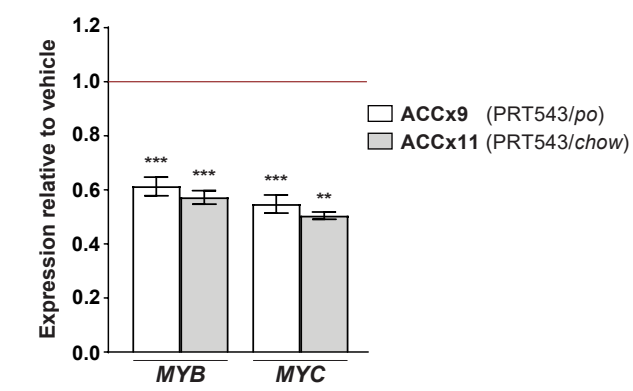

Supplementary Figure 5

Supplement: Supplementary file 5 — Supplementary Figure 5: (A) ACCx9 and ACCx11 tumor tissues were harvested from PRT543 or vehicle treated mice, lysates were collected and analyzed by western blot for the expression of SmD3. β-actin was used as loading control. (B) RT-PCR results demonstrating significant reduction of MYB and MYC gene expression in PRT543 treated ACCx9 and ACCx11 PDX models relative to the control animals (red line) [file 13046_2024_3270_MOESM5_ESM.pdf]

**A.**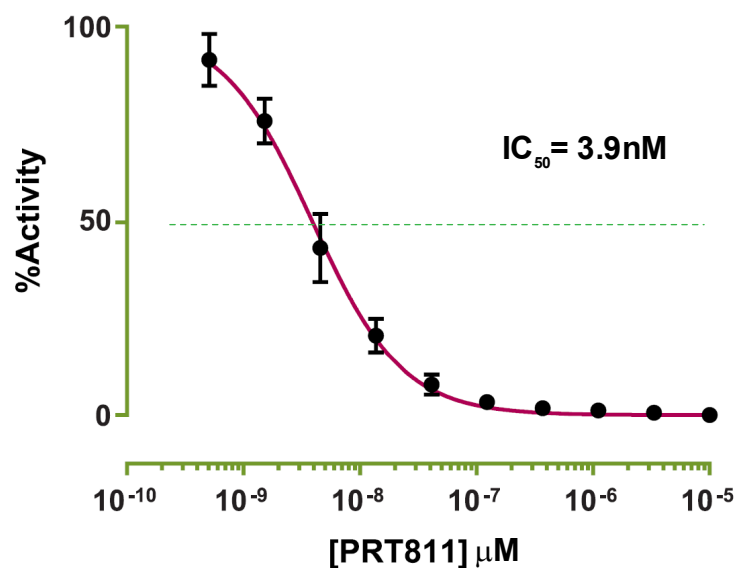**B.**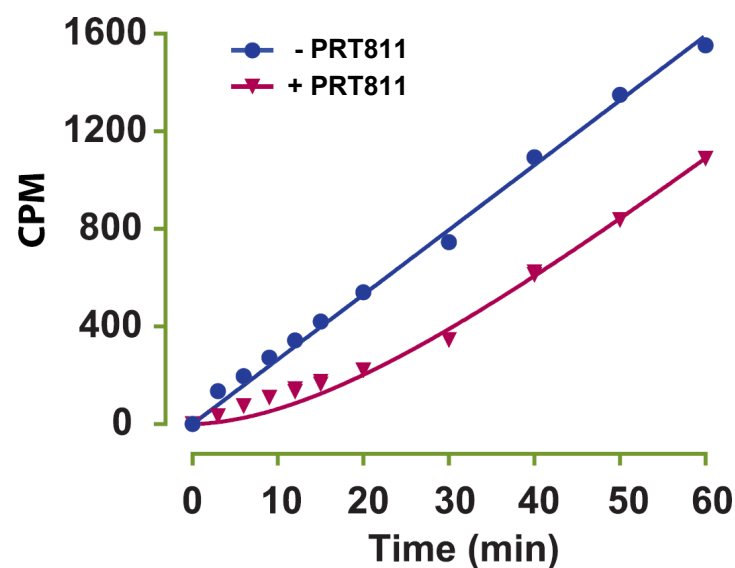**C.**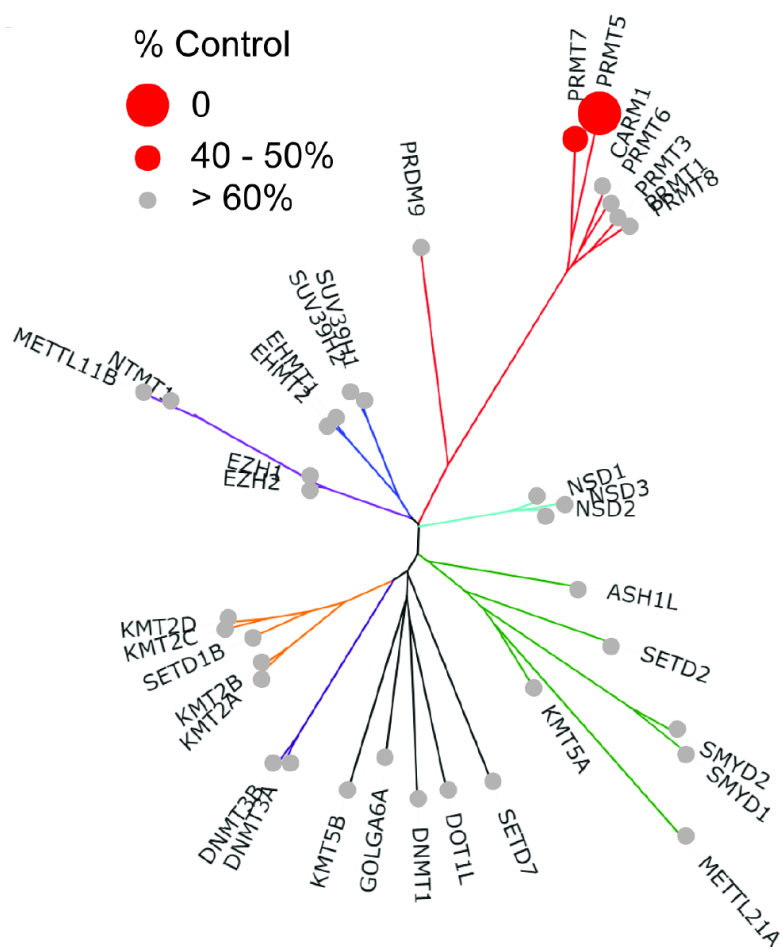

**Supplementary Figure 6**

Supplement: Supplementary file 6 — Supplementary Figure 6: (A) Concentration-dependent inhibition of PRTMT5/MEP50 enzymatic activity by PRT811. (B) Jump dilution assay showing PRMT5/MEP50 enzymatic progress curve in the absence and presence of PRT811. CPM - counts per minute. (C) Biochemical selectivity of 10µM PRT811 against 37 human methyltransferases. Percent control represents % enzymatic activity remaining in the presence of PRT811 relative to DMSO control [file 13046_2024_3270_MOESM6_ESM.pdf]

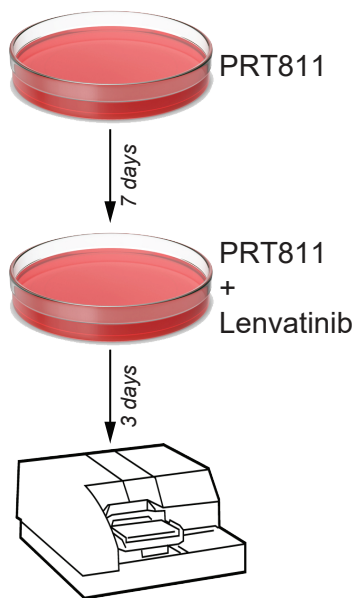

**Supplementary Figure 7**

Supplement: Supplementary file 7 — Supplementary Figure 7: Schematic presentation of the experiment. HACC2A and UFH2 cell lines were treated with the IC50 concentrations of PRT811 for 7 days, following by the exposure to IC50 concentrations of lenvatinib at days 8–10 [file 13046_2024_3270_MOESM7_ESM.pdf]
